# An overview of model uncertainty and variability in LLM-based sentiment analysis: challenges, mitigation strategies, and the role of explainability

**DOI:** 10.3389/frai.2025.1609097

**Published:** 2025-08-11

**Authors:** David Herrera-Poyatos, Carlos Peláez-González, Cristina Zuheros, Andrés Herrera-Poyatos, Virilo Tejedor, Francisco Herrera, Rosana Montes

**Affiliations:** ^1^Department of Computer Science and Artificial Intelligence, Andalusian Institute of Data Science and Computational Intelligence (DaSCI), University of Granada, Granada, Spain; ^2^Department of Software Engineering, Andalusian Institute of Data Science and Computational Intelligence (DaSCI), University of Granada, Granada, Spain

**Keywords:** sentiment analysis, large language models, uncertainty, model variability problem, LLM-based sentiment analysis

## Abstract

Large Language Models (LLMs) have significantly advanced sentiment analysis, yet their inherent uncertainty and variability pose critical challenges to achieving reliable and consistent outcomes. This paper systematically explores the Model Variability Problem (MVP) in LLM-based sentiment analysis, characterized by inconsistent sentiment classification, polarization, and uncertainty arising from stochastic inference mechanisms, prompt sensitivity, and biases in training data. We present illustrative examples and two case studies to highlight its impact and analyze the core causes of MVP, discussing a dozen fundamental reasons for model variability. We pay especial atenttion to explainabily, with an analysis of its importance in LLMs from the MVP perspective. In addition, we investigate key challenges and mitigation strategies, paying particular attention to the role of temperature as a driver of output randomness and highlighting the crucial role of explainability in improving transparency and user trust. By providing a structured perspective on stability, reproducibility, and trustworthiness, this study helps develop more reliable, explainable, and robust sentiment analysis models, facilitating their deployment in high-risk domains such as finance, healthcare and policy making, among others.

## 1 Introduction

Sentiment analysis has emerged as a critical application of large language models (LLM) in fields such as customer feedback analysis, financial market predictions, brand reputation monitoring, and trend detection on social media. In many of these domains—e.g., algorithmic trading or automated credit scoring—even a few-tenth shift in polarity can move millions of dollars or trigger high-impact decisions, so robustness and consistency are essential. Prompt-based LLMs are especially attractive in such settings because, unlike traditional classifiers that require full retraining, their behavior can be redirected instantly through natural-language prompts, allowing domain experts to adjust sentiment criteria as regulations, market language, or risk tolerances evolve. Traditional sentiment analysis approaches relied on rule-based lexicons or supervised machine learning models, which, while interpretable, struggled with nuanced expressions such as sarcasm, irony, and contextual sentiment shifts (Wankhade et al., 2022; Zhang et al., 2024; Krugmann and Hartmann, 2024). With the introduction of LLMs such as GPT-4, BERT, RoBERTa, and T5, sentiment classification has improved significantly in terms of precision, contextual understanding, and adaptability to various domains. LLMs leverage their vast pretraining corpora and deep transformer architectures to understand sentiment beyond simple polarity detection, incorporating emotion classification, aspect-based sentiment analysis, and entity-level sentiment extraction (Yang H. et al., 2024).

Despite these advancements, the reliance on probabilistic text generation and deep feature representations introduces challenges related to output variability, inconsistency between inference runs, and susceptibility to biases in training data (Beigi et al., 2024). Unlike traditional classifiers, which yield deterministic output, LLMs can generate different sentiment scores for the same input based on factors such as decoding parameters, prompt phrasing, and the model's internal confidence in its predictions. This variability is particularly concerning in high-risk decision-making applications, such as automated financial sentiment analysis, where unstable predictions can lead to inaccurate market forecasts. Addressing this issue requires robust techniques such as uncertainty quantification, model calibration, and ensemble averaging to enhance stability, reliability, and explainability in sentiment classification.

The Model Variability Problem (MVP) refers to the phenomenon in which an LLM or machine learning system produces inconsistent outputs for the same input in multiple runs (Wankhade et al., 2022). This issue arises in various natural language processing applications but is particularly problematic in sentiment analysis, where a model tasked with assigning a sentiment polarity score (ranging from 0 to 1) may yield different values for identical input text. These inconsistencies result from the stochastic nature of LLM inference mechanisms, leading to fluctuations that impact the reliability, trustworthiness, and downstream applications of the model in decision-making systems.

The survey (Wankhade et al., 2022), entitled “*A Survey on Sentiment Analysis Methods, Applications, and Challenges,”* pay attention to the uncertainty and the MVP. It aligns closely with the uncertainty and variability described in the context of LLM-based sentiment analysis. The authors discuss key challenges such as domain dependency, ambiguity in textual data, implicit language understanding (including sarcasm and irony), and feature selection complexities—all contributing factors to variability issues observed in modern sentiment analysis approaches. These identified challenges echo the broader MVP, highlighting fundamental issues like aleatoric uncertainty due to ambiguous language and epistemic uncertainty caused by insufficient domain knowledge or lack of generalization capabilities in models. In addition, the survey emphasizes the limitations of conventional sentiment analysis methods, including lexicon-based and supervised approaches, stressing that each method faces difficulties in reliably capturing nuanced sentiment, particularly in real-world settings involving sarcasm, irony, slang, and domain-specific terminology. This is directly related to MVP, as similar ambiguities significantly affect LLM predictions, causing inconsistent sentiment classifications between different inference runs (Da et al., 2025).

Recent advances in LLMs have significantly impacted sentiment analysis, notably enhancing sentiment analysis-based crowd decision making (ESA-CDM) by using structured prompt design strategies, as evidenced by recent empirical studies utilizing different LLMs (Herrera-Poyatos et al., 2025). Prompt-based approaches are especially attractive in such contexts because they can be adapted by domain experts without costly re-training, provide an interpretable record of decision rules, and complement traditional fine-tuned classifiers when rapid domain shifts occur. Although these methods show promising potential for extracting consensus-driven sentiment classifications from large-scale opinion datasets, the inherent uncertainty and variability within LLMs pose fundamental challenges. The variability problem, which arises from sensitivity to prompt variations, stochastic inference methods, and biases of training datasets, critically impacts the reliability and consistency of crowd-driven sentiment classification. This paper extends the existing analysis by exploring the uncertainty and variability factors that affect the accuracy of sentiment classification, highlighting the need for refined methodologies that address consistency, robustness, and transparency in ESA-CDM contexts supported by LLM.

In addition, given the high stakes involved in ESA-CDM and other critical applications of sentiment analysis, particular emphasis must be placed on the explainability and reliability of LLM-generated sentiment predictions and explanations. As discussed extensively in the recent literature, the opaque nature of LLMs impedes understanding of model predictions, posing significant barriers to trust and user acceptance. Herrera (2025) underscores the importance of adopting explainability frameworks to improve the transparency of AI-driven sentiment analysis, facilitating better human-AI interaction, trust building, and informed decision-making. Thus, this paper also explores strategies to integrate robust explainability methodologies, ensuring that sentiment classification outputs are not only consistent and reliable but also transparent and comprehensible for diverse stakeholders involved in ESA-CDM contexts.

The consequences of model variability in sentiment analysis and other natural language processing applications include:

- Unstable sentiment classification: a business analyzing customer feedback may receive conflicting sentiment scores from the same input.
- Bias amplification: variability can exacerbate inherent model biases, leading to systematic errors in human-AI decision making, where AI systems must assist the human with advice.
- Reduced reproducibility in sentiment analysis with LLMs: studies relying on LLM output may not reproduce results, affecting model benchmarking.
- Challenges in trustworthy AI: end users and policymakers require an explainable and consistent AI behavior, which variability undermines.

The purpose of this paper is to provide a comprehensive and holistic analysis of MVP and associated uncertainties that arise from the use of LLM-based sentiment analysis. We pay attention to challenges, mitigation strategies, and the role of explainability.

We adopt a structured approach from the MVP perspective, beginning with some illustrative examples to show the variability in LLM-generated sentiment predictions. Subsequently, we performed an in-depth analysis of the primary factors contributing to uncertainty and variability, focusing on critical aspects such as stochastic inference methods, prompt sensitivity, and biases in training data. Finally, the article identifies emerging trends and key challenges and describes promising directions and methodologies as mitigation strategies.

In addition, we also pay attention to a fundamental aspect of AI systems in general and LLMs in particular—the AI explainability. Explainable AI (XAI) allows us to get information on the internal mechanisms, reasoning pathways, and decision-making processes of models. XAI is very important from the point of view of trust AI (Afroogh et al., 2024). As discussed in Herrera (2025), explainability is not only a technical challenge but also a human-centered and philosophical endeavor, essential to foster meaningful interaction and accountability in human-AI ecosystems. As highlighted in Luo and Specia (2024), XAI in LLMs must evolve from mere understanding of model behavior to practical utilization, where explanations serve real-world user needs in dynamic and uncertain contexts. In the context of sentiment analysis and model variability, XAI can provide an essential lens through which uncertainty and trust can be assessed, contested, and aligned with user expectations.

In order to develop the discussion and analysis, the paper is organized as follows. Section 2 shows some illustrative examples on the problem of focus, uncertainty, and variability. Section 3 introduces the fundamental reasons for model variability with a literature analysis. Section 4 introduces a reflection and analysis on the importance of explainability for LLM. Section 5 discusses challenges and mitigation strategies for MVP in LLM-based sentiment analysis. Finally, some conclusions are pointed out in Section 6.

## 2 Case studies and illustrative examples to show the uncertainty and model variability

It has interest to present examples when addressing uncertainty and MVP in LLM-based sentiment analysis, as tangible demonstrations facilitate clearer visualization and a deeper understanding of this complex phenomenon. Real-world examples offer an accessible entry point for grasping abstract concepts such as stochastic inference, prompt sensitivity, or subtle contextual shifts, enabling more effective discussions, diagnostics, and ultimately the formulation of robust mitigation strategies.

We present two case studies in Section 2.1 and Section 2.2 that illustrate the unpredictable nature of model output when dealing with real-world sentiment data. In the first case study, we explore the variability in repeated sentiment evaluations using the GPT-4o model, showing how the same input can produce fluctuating predictions due to stochastic inference. In the second case study, we analyze inconsistencies between numerical sentiment scores and categorical labels obtained from the Mixtral 8x22B model, revealing the impact of contextual interpretation on model reliability.

Following these case studies, we discuss the broader implications of model variability through additional illustrative examples in Section 2.3. These examples further emphasize how unpredictable and inconsistent sentiment predictions can undermine the credibility of LLM-based applications, particularly in contexts such as customer reviews and finance.

We present both empirical evidence and conceptual scenarios to foster a deeper understanding of the critical challenges posed by uncertainty in sentiment analysis. Our aim is to advocate robust strategies to mitigate model variability and enhance trustworthiness.

### 2.1 Case study 1: TripR-2020Large dataset

In order to introduce a short analysis, we consider the TripR-2020Large dataset (Zuheros et al., 2021) since it collects real data to evaluate Decision-Making (DM) models with unrestricted natural language input. The TripR-2020Large dataset^1^ contains 474 written reviews in English from 132 TripAdvisor users, who express their experiences in four restaurants, forming the set of alternatives: *X* = {*x*_1_, *x*_2_, *x*_3_, *x*_4_} = {*The Oxo Tower, The Wolseley, The Ivy, J. Sheekey*}. Since not all experts review every restaurant, the dataset comprises slightly fewer than 132 × 4 documents.

**Figure 1:**
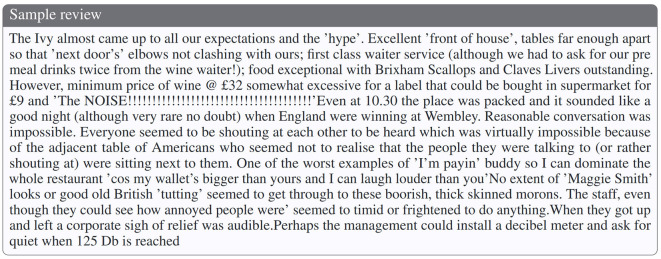
Review from the TripR-2020Large dataset.

In the following, we present an analysis of the opinion introduced in Figure 1 and show the uncertainty observed when the query is repeated 100 times using the GPT 4o model, accessed through the OpenAI API. All other decoding parameters (e.g., top-p = 1.0) were left at their default values; only the temperature was varied, 1.0 for the stochastic setting and 0.0 for the near-deterministic one. Some options, such as top-k, are not exposed through the API and therefore cannot be modified, while others—like seed—are still in Beta; all were kept at their defaults. This analysis highlights how variability manifests itself in model predictions and quantifies the inherent uncertainty associated with sentiment analysis. To conduct this study, we used the following prompt to assess the sentiment of the given review:

Rate the sentiment of this review on a continuous scale from 0 to 1, where 0 means entirely negative, and 1 means entirely positive. The answer must be only a number: [DOCUMENT]

**Figure 2:**
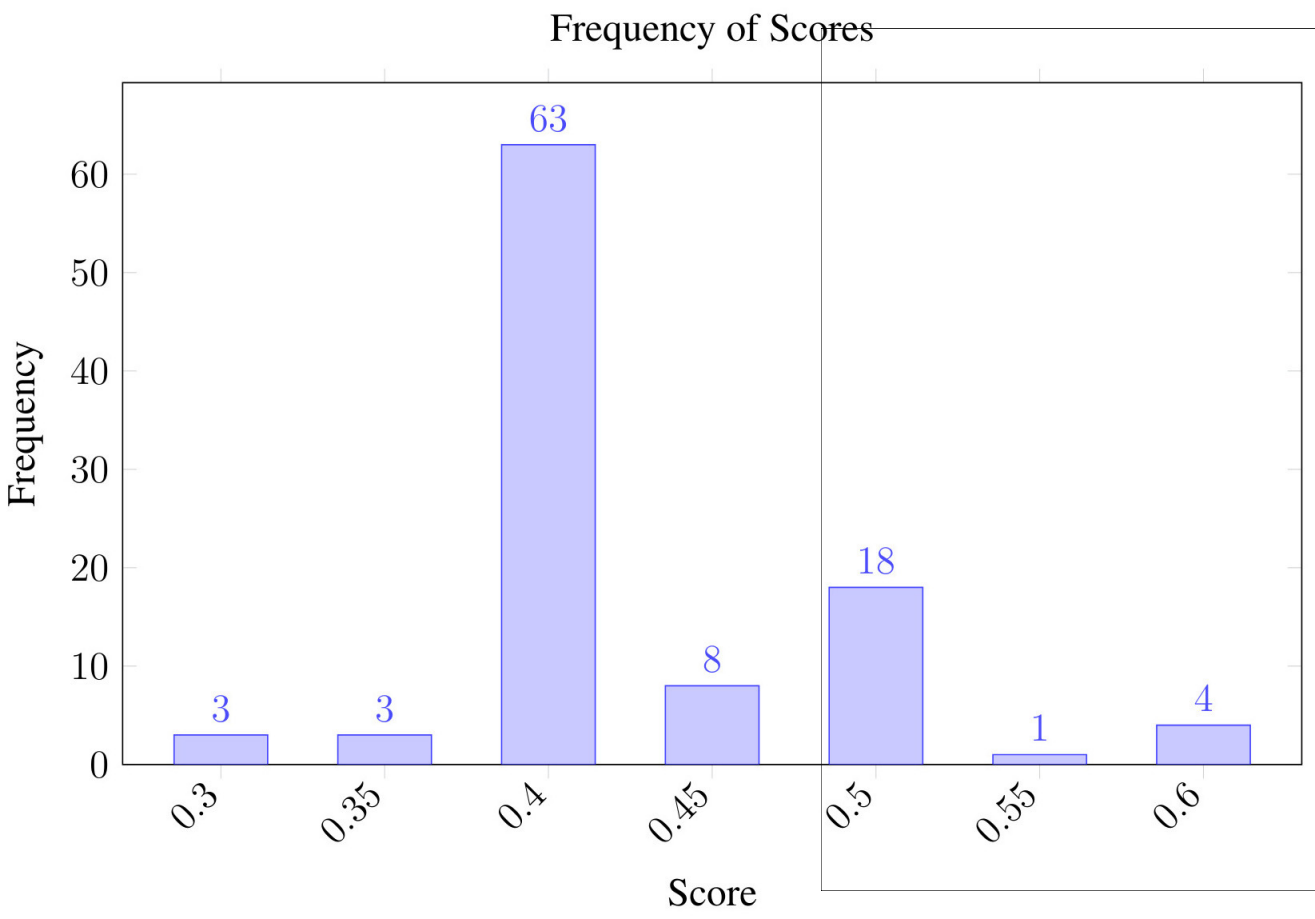
Uncertainty under the opinion analysis using GPT 4o with temperature 1.

**Figure 3:**
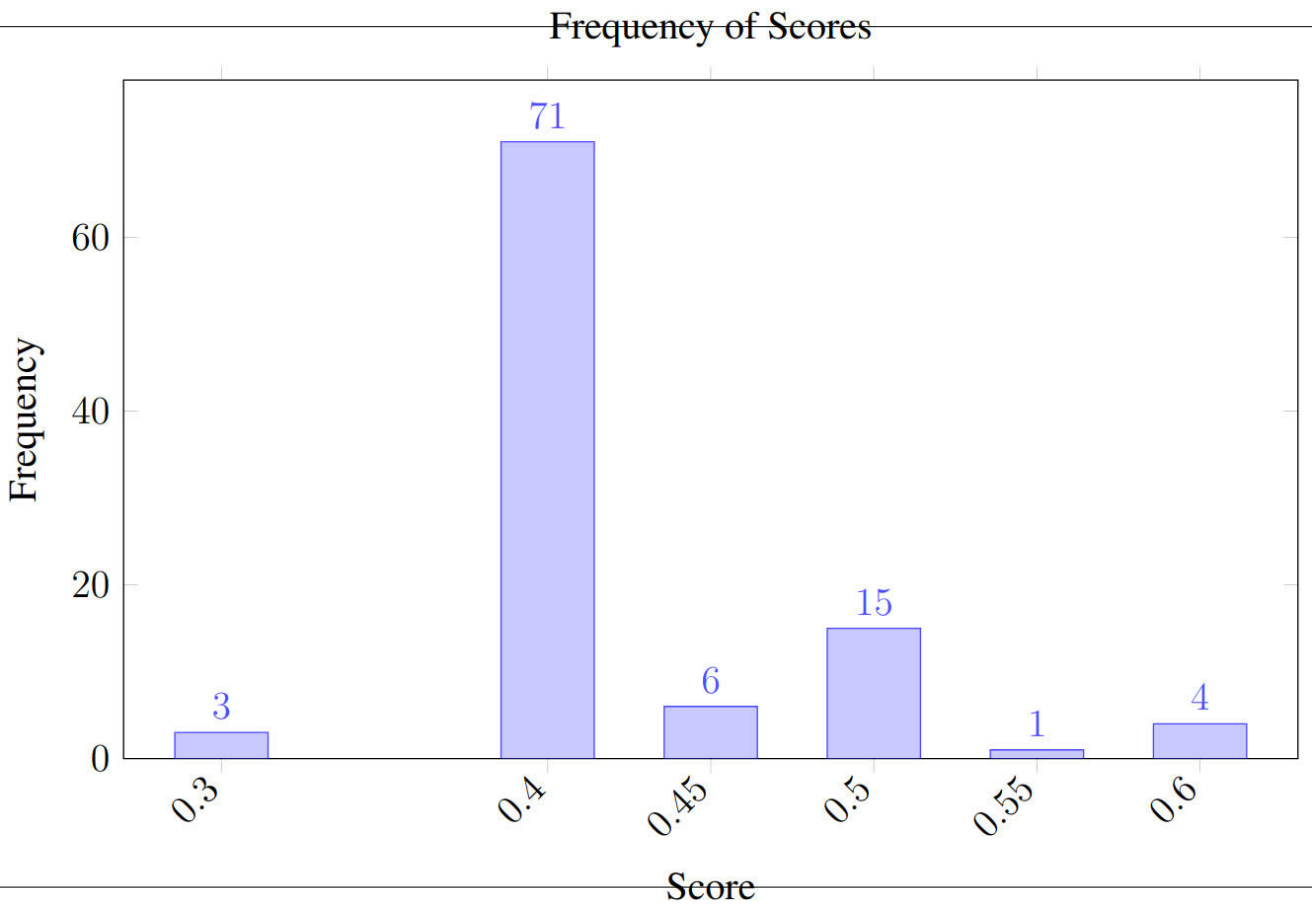
Uncertainty under the opinion analysis using GPT 4o with temperature 0.

The resulting variability distribution, shown in Figure 2, shows how the predictions (polarity) fluctuate even when analyzing the same review multiple times while using a temperature of 1.0. It should be noted that polarity fluctuates between negative (0.3) and positive (0.6) values. Meanwhile, Figure 3 shows the uncertainty when the temperature is set to 0.0, in theory, reducing its stochastic behavior. The scores still span the same range—0.3 (negative) to 0.6 (positive)—as in the temperature 1.0 run, but with temperature 0.0, 71% of the outputs collapse to 0.4, compared with 63% under temperature 1.0.

Despite the narrower histogram, the extreme scores (0.3 and 0.6) still occur, so the possible polarity swing for downstream decisions remains unchanged.

### 2.2 Case study 2: global inconsistency on sentiment analysis problem

**Figure 4:**
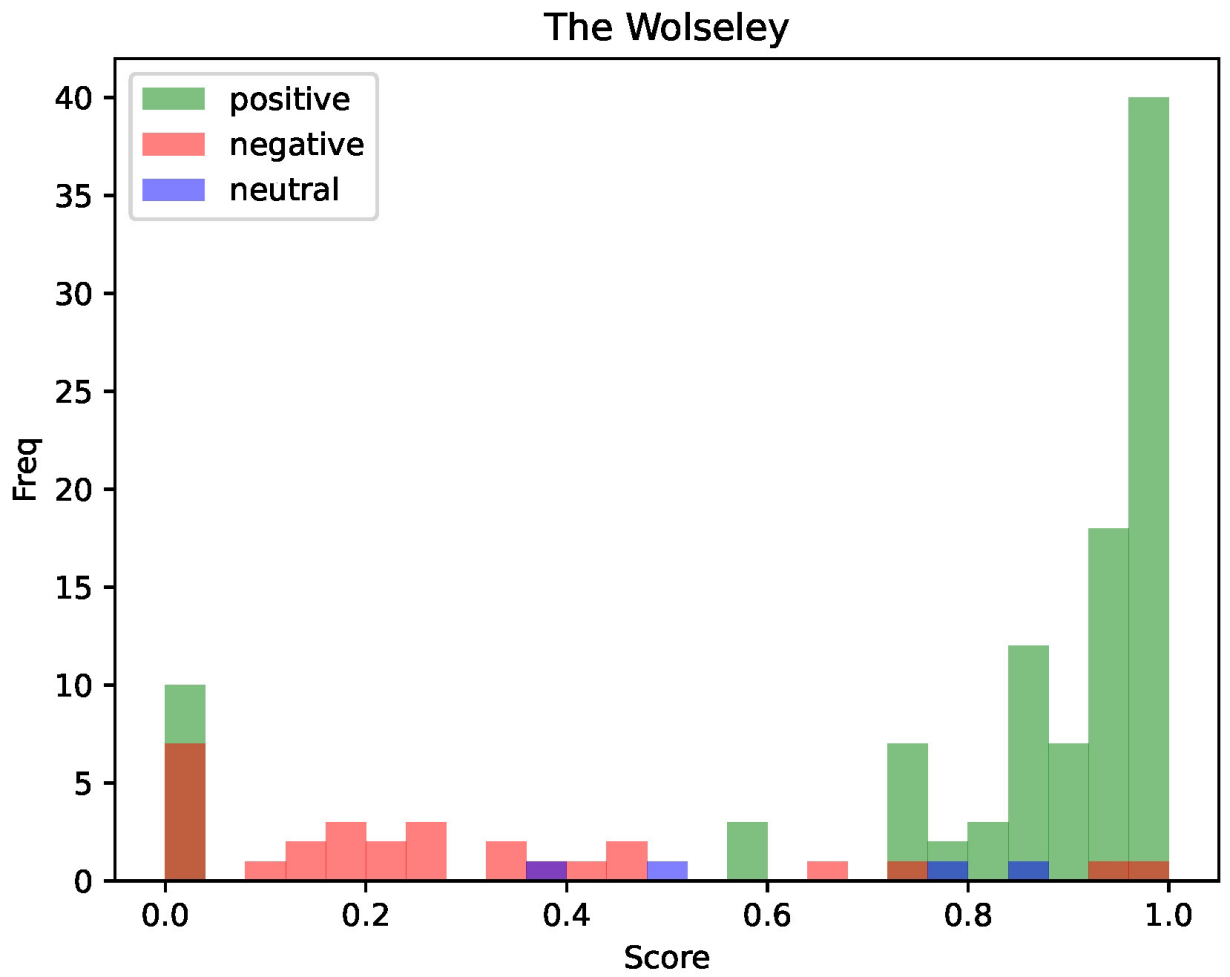
Inconsistency between numerical and linguistic polarity using Mixtral 8x22B model.

The second study, shown in Figure 4, presents the evaluation of all opinions for the restaurant *The Wolseley*, obtained using the Mixtral 8x22B model. The model was served locally via llama.cpp,^2^ a lightweight, dependency-free C/C++ framework for efficient LLM inference across diverse hardware platforms, on four H100 GPUs with 80 GB VRAM each; parameters were kept at their documented defaults: temperature = 1.0, top-p = 1.0, top-k = 40 and the random seed flag was left unset. This study aims to analyze the consistency between numerical sentiment scores and categorical labels, highlighting the inherent challenges posed by prompt sensitivity and contextual variability.

The data shown in Figure 4 consists of the following elements:

- The histogram illustrates the frequency of sentiment scores ranging from 0.0 (entirely negative) to 1.0 (entirely positive), with intermediate values indicating varying degrees of sentiment polarity.
- The color of each bar represents the sentiment label predicted by the same model, obtained by using a different prompt that explicitly asks for a label among *positive, neutral* or *negative*. Therefore, the color coding reflects the prediction of the sentiment label, while the height of each bar corresponds to the frequency of scores.

The histogram is generated by combining the results from two different prompts, which are as follows:

Classify the sentiment of the following text as positive, neutral or negative, the answer must be a single label and one word: [DOCUMENT]

Classify the sentiment of the following text using a score between 0 and 1, where 0 represents a completely negative sentiment and 1 represents a completely positive sentiment. The answer must be only a number: [DOCUMENT]

The responses obtained from these prompts are then matched for each review, allowing both the model's score and label predictions to be visualized together in the histogram.

Figure 4 reveals significant inconsistencies between the numerical sentiment score and the categorical label. For example, some reviews classified as negative exhibit relatively high sentiment scores, while positive labels occasionally appear at low scores. This discrepancy highlights the model's variability, reflecting challenges related to stochastic inference and prompt sensitivity.

The two presented case studies clearly demonstrate the profound challenges posed by uncertainty and model variability in LLM-based sentiment analysis. The first study highlights the inherent instability of model outputs when repeating the same sentiment analysis query multiple times, revealing how even minor stochastic variations can lead to significantly different predictions. This phenomenon is particularly concerning when consistent input should logically yield stable output, but the inherent randomness of the model results in a fluctuating range of sentiment scores.

The second study exposes an equally critical issue: the inconsistency between numerical sentiment scores and categorical labels. Even when using the same model and evaluating the same input with slightly different prompts, the outputs diverge significantly, revealing a lack of coherence between quantitative and qualitative sentiment assessment. This inconsistency points to a fundamental challenge in how LLMs interpret and classify sentiment, especially when prompt phrasing subtly alters the context or interpretation.

Together, these case studies reveal that model variability and inconsistency are not merely occasional glitches, but systematic challenges that arise from the nature of LLM-based sentiment analysis. This variability significantly undermines the reliability and trustworthiness of automated sentiment classification, particularly in critical applications such as customer feedback analysis, healthcare monitoring, and financial sentiment prediction.

By quantifying the extent of variability and demonstrating its impact through real-world examples, we highlight the urgent need for robust mitigation techniques that can reduce the unpredictability of model outputs and enhance the stability of sentiment predictions in practical applications.

### 2.3 Illustrative examples

Illustrative examples can vividly showcase the practical consequences of model variability, particularly in high-risk domains such as finance, healthcare, and consumer analytics, among others. When variability is demonstrated through clear cases, such as analyzing sentiment from customer reviews or interpreting financial news headlines, users and developers alike can better appreciate its impact on reliability and trustworthiness. This practice not only helps to recognize the urgent need for stable and transparent AI solutions but also emphasizes the importance of investing in research to develop techniques for reducing MVP, thus enhancing the overall dependability and effectiveness of sentiment-analysis applications powered by LLMs. We now introduce three illustrative examples that demonstrate the real-world implications of model variability.

#### 2.3.1 Sentiment variability in customer reviews

To clearly illustrate the phenomenon of model variability in sentiment analysis, consider an example involving customer reviews analyzed by GPT-4. When analyzing the following sentence.

“*Oh great, another rainy day!”*

GPT-4 may interpret this sentiment differently in multiple inference runs. In one instance, influenced by the literal meaning of “*great,”* it might assign a neutral or slightly positive sentiment score, whereas in another instance, detecting potential sarcasm, it could produce a strongly negative sentiment. Such inconsistencies reflect the inherent uncertainty that stems from the ambiguity of natural language and the stochastic decoding processes employed by LLMs, significantly affecting the reliability of sentiment classifications in practical, real-world applications.

#### 2.3.2 Variability in financial sentiment analysis

Another illustrative example is observed in financial sentiment analysis tasks using LLMs such as ChatGPT, where market sentiment predictions based on news headlines exhibit considerable variability. For example, the following headline might trigger diverse sentiment polarity scores across different runs, ranging from cautiously optimistic to highly positive.

“*Company X announces a surprise merger”*

This variation arises due to differences in prompt construction, subtle contextual interpretations, and model parameter settings such as temperature or top-k sampling, leading to instability that critically impacts decision-making in scenarios requiring precision and reproducibility, such as algorithmic trading or risk assessment. These examples underscore the urgent need to understand and mitigate MVP to improve trust and effectiveness in LLM-based sentiment analysis.

#### 2.3.3 Hypothetical high-risk finance scenario

Consider a realistic trading scenario. An investment-bank dashboard ingests live news wires and relies on an LLM-based sentiment module to trigger automated “buy,” “hold,” or “sell” signals.

“*Central bank hints at surprise rate cut next quarter”*

**Figure 5:**
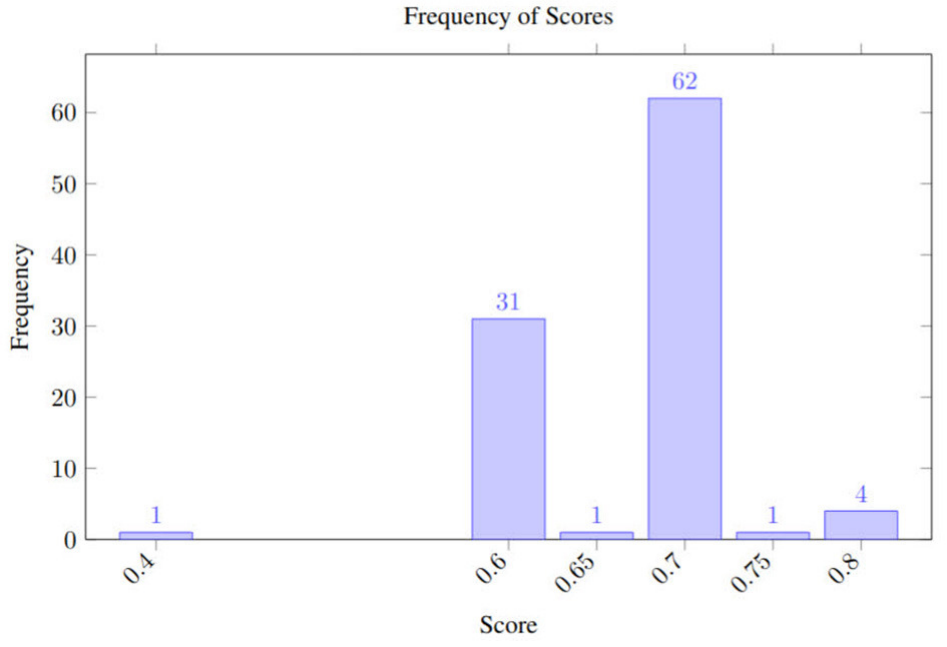
Uncertainty for the hypothetical high-risk scenario using GPT 4o with temperature 1.

To show a hypothetical experiment, we fed gpt-4o the hypothetical headline one hundred times through the OpenAI API, using the same parameters as in our earlier TripR experiment: temperature = 1.0, top-p = 1.0; all options were left at their defaults. The resulting sentiment scores, visualized in Figure 5 ranged from 0.40 to 0.80 with a mean of 0.67. Under a typical rule that fires a *sell* for scores below 0.4, a *hold* between 0.4 and 0.6, and a *buy* for scores above 0.6. The experiment shows that the same headline could trigger different trading actions. This kind of inconsistency can expose the trading desk to significant intraday risk and possibly draw regulatory attention.

This hypothetical yet reproducible example shows why stability-aware evaluation is indispensable in finance: reporting only an average score would have concealed a potentially costly vulnerability.

## 3 A dozen fundamental reasons for model variability

MVP refers to the phenomenon where LLMs produce inconsistent outputs for the same input on multiple runs. Based on analysis of key literature, we identify a dozen fundamental reasons that contribute to this issue. We provide a short introduction to each and the appropriate literature that supports them.

### 3.1 Aleatoric and epistemic uncertainty

Uncertainty in sentiment classification can be categorized into two primary types: aleatoric uncertainty, which arises from inherent randomness in data, and epistemic uncertainty, which stems from knowledge limitations within the model. These factors contribute significantly to MVP, leading to inconsistent sentiment predictions in inference runs.

Aleatoric uncertainty manifests itself when textual data contains ambiguities, sarcasm, or sentimentally mixed expressions, making the interpretation highly dependent on context. Sentiment analysis models often struggle with these complexities, leading to unstable and inconsistent sentiment classifications. Addressing aleatoric uncertainty requires enhanced contextual embeddings, advanced linguistic modeling techniques, and probabilistic output representations to better handle ambiguous textual inputs. Studies such as Shorinwa et al. (2024) identify data-driven noise—stemming from annotation inconsistencies, ambiguous labels, and linguistic variability—as a primary source of aleatoric uncertainty in LLM-based sentiment analysis. Furthermore, Beigi et al. (2024) highlights how social media slang, domain shifts, and informal text variations exacerbate this uncertainty, making it challenging for models to generalize sentiment classification across different contexts.

Epistemic uncertainty, on the other hand, arises when gaps in pre-training data prevent the model from confidently handling unfamiliar or underrepresented linguistic structures. This type of uncertainty leads to unstable predictions, particularly in domain-specific sentiment tasks where LLMs lack sufficient exposure to nuanced vocabulary. Furthermore, epistemic uncertainty can result in confidence misalignment, where models express unwarranted certainty in incorrect predictions, undermining trustworthiness. Reveilhac and Morselli (2024) explores how knowledge limitations in LLM-powered voting systems introduce contradictions in sentiment-based decision-making, reinforcing the need for uncertainty-aware training approaches. Similarly, Passerini et al. (2025) shows how human-LLM interactions can mitigate or amplify epistemic uncertainty, depending on whether the model is trained on high-quality, diverse sentiment data or exposed to biased, repetitive user input.

Addressing both aleatoric and epistemic uncertainty requires a multifaceted approach, integrating data augmentation techniques, uncertainty-aware learning frameworks, and structured fine-tuning methodologies. By enriching training data, improving contextual sensitivity, and implementing confidence calibration techniques, LLMs can achieve greater stability and reliability in sentiment classification. Future research must focus on quantifying these uncertainties systematically and designing adaptive models that can dynamically adjust confidence levels based on input complexity.

### 3.2 The role of temperature in the variability of LLM output

One of the most influential hyperparameters yet examined in LLM is the *temperature*, which directly controls the stochasticity of the output generation process. Temperature scales the logits (output probabilities) before applying the softmax function, thus modulating how deterministic or exploratory the model's sampling behavior becomes during inference. Lower temperatures (e.g., *T* = 0.1–0.3) make the model output more deterministic by increasing the probability mass on the most likely tokens, while higher temperatures (e.g., *T* = 0.8–1.5) introduce more randomness, promoting diversity and creativity at the expense of consistency.

This parameter has critical implications for the MVP. High-temperature settings, although useful in open-ended tasks like creative writing or brainstorming, inherently increase output variance—even for semantically equivalent prompts. This introduces unpredictability and reduces reliability in use cases where stability, reproducibility, and fairness are essential, such as sentiment analysis, medical decision support, or legal QA. In such contexts, repeated queries with identical prompts may yield divergent responses, undermine user trust, and compromise decision integrity.

Recent studies have shown that even at moderate temperature settings (*T* = 0.7), LLMs such as GPT-3.5, Falcon, or LLaMA exhibit significant variance in sentiment polarity, justification styles, and factuality levels (Beigi et al., 2024). This variance becomes particularly problematic in applications relying on aggregate decision models (e.g., crowd decision-making or sentiment voting systems), where fluctuations in individual model predictions can distort final consensus or rankings. Moreover, the interaction between temperature and prompt sensitivity exacerbates MVP: small syntactic rephrasings can drastically shift the model sampling trajectory under high-temperature decoding.

Understanding and controlling the effects of temperature is thus vital not only for task-specific performance but also for broader goals in LLM trustworthiness, explainability, and reproducibility.

### 3.3 Inference stochasticity and sampling mechanisms

MVP in LLM-based sentiment analysis is exacerbated by stochastic inference mechanisms that introduce nondeterministic behavior into sentiment predictions. LLMs employ stochastic decoding strategies, including temperature scaling (previously analyzed) and top-k sampling, which introduce variability into their responses; deterministic methods such as beam search are also available and, when used with fixed model parameters and decoding settings, do not contribute to this stochasticity.

Although these methods improve response diversity and adaptability, they also lead to inconsistent sentiment output, even when processing identical inputs multiple times. This variability poses significant challenges in applications that demand stability and reproducibility, such as financial sentiment analysis, legal document evaluation, and automated decision-making systems.

One of the main contributors to sentiment variability in LLMs is the randomness of token selection during inference, as evidenced in studies such as (Ye et al., 2024). The authors analyze how sampling randomness impacts sentiment classifications, demonstrating that identical sentiment analysis tasks can yield inconsistent results due to stochastic decoding. The study specifically examines temperature scaling and top-k sampling, highlighting how these hyperparameters influence the distribution of possible sentiment labels. Similarly, Lefort et al. (2024) identifies quantile-based variations in sentiment classification across repeated runs, further confirming that LLM inference introduces an inherent degree of unpredictability into sentiment analysis.

Furthermore, research by Loya et al. (2023) explores how hyperparameter sensitivity impacts model decision making, emphasizing that even when inference settings are held constant, minor prompt variations can still lead to differing sentiment classifications. This highlights an essential issue: stochastic variability is not just a function of temperature or top k sampling, but also depends on contextual prompt dependencies, making the problem even more complex. The findings of Atil et al. (2024) further validate this concern, demonstrating accuracy fluctuations of up to 10% across repeated identical inference runs, even in cases where deterministic configurations were enforced. Their study introduces stability-focused evaluation metrics, including the Total Agreement Rate at N (TARr@N and TARa@N), which systematically measure inference instability across different sentiment classification tasks.

Beyond stochastic inference, uncertainty quantification techniques play a crucial role in mitigating variability in sentiment classification. As highlighted in Ji et al. (2025), epistemic and aleatoric uncertainty significantly impact the trustworthiness of LLM sentiment predictions. The study proposes uncertainty estimation based on entropy, semantic consistency checks, and confidence-aware calibration techniques, which help mitigate inconsistencies by quantifying and adjusting model confidence in uncertain sentiment classifications. These statistical methodologies are especially relevant in high-risk sentiment-driven applications, where even slight variations in LLM sentiment output could have significant financial, legal, or societal implications.

To counteract the effects of inference stochasticity and enhance stability in sentiment classification, recent studies propose multiple mitigation strategies. Atil et al. (2024) suggests integrating ensemble-based averaging techniques, which aggregate the outputs of multiple inference runs to reduce variability and reinforce the stability of classification. Similarly, confidence calibration techniques, as explored in Ye et al. (2024), provide mechanisms to align model predictions with uncertainty quantification, reducing the probability of sentiment fluctuations due to stochastic effects. These findings reinforce the need for systematic stability-aware evaluation frameworks in sentiment analysis, ensuring that LLM-based models maintain consistency and reliability in real-world applications.

### 3.4 Bias, scale, and multimodal inconsistencies in LLM-based sentiment classification

MVP in LLM-based sentiment analysis is deeply influenced by a combination of factors including model scale, sentiment label bias, architectural discrepancies across model versions, and multimodal input conflicts. These interconnected sources of variability compromise the consistency, interpretability, and trustworthiness of sentiment classification systems, particularly in high-risk domains.

On the one hand, larger LLMs typically achieve superior linguistic performance, but they are also more prone to overfitting on noisy or biased pretraining data, amplifying sentiment prediction instability. Ye et al. (2024) demonstrates that as model size increases, so does variance in sentiment output, particularly in the presence of ambiguous or nuanced expressions like sarcasm. This makes larger models more sensitive to prompt phrasing and input context shifts, despite their enhanced representational capacity. Simultaneously, Shorinwa et al. (2024) identifies sentiment label bias in training data as a key driver of systematic errors. When sentiment categories or linguistic styles are unevenly distributed, models tend to inherit and amplify those patterns, leading to skewed or unreliable sentiment predictions.

These challenges are compounded by considering bias and variability between LLM versions and between modalities. As Krugmann and Hartmann (2024) and Zhang et al. (2024) highlight, different LLM variants (e.g., GPT-3.5 vs. GPT-4) often produce divergent sentiment classifications for identical inputs due to architectural differences and varied fine-tuning protocols. Such inconsistencies compromise the reproducibility and comparability of the model. Furthermore, Yang H. et al. (2024) reveals how multimodal sentiment conflicts, especially between textual and visual data, can result in conflicting sentiment interpretations when LLMs do not align nonverbal signals with textual content. For example, a sarcastic tweet paired with a cheerful image may be misclassified due to a misalignment of the modality.

To address these intertwined issues, a multifaceted mitigation strategy is required. First, bias-aware training and dataset balancing must be prioritized to reduce inherited skew. Second, uncertainty quantification (e.g., Bayesian modeling, temperature scaling) can help assess prediction reliability. Third, cross-version calibration protocols should be adopted to harmonize outputs across LLM releases, while multimodal alignment techniques (e.g., cross-modal attention tuning, sentiment fusion models) are necessary to ensure coherent sentiment interpretation across inputs.

In conclusion, MVP in sentiment analysis arises not only from model complexity and label bias but also from version-specific discrepancies and multimodal misalignments. Resolving these challenges will require integrated debiasing, stability-aware training, and robust alignment frameworks that collectively support consistent and interpretable sentiment predictions across models and media types.

### 3.5 Implicit spectral instability and self-regularization failure in LLMs

Recent advances by Martin and Mahoney (2021) and Martin et al. (2021) uncover a fundamental source of model variability rooted in the spectral properties of neural network weight matrices. Their work demonstrates that deep models—including LLMs—exhibit an *implicit self-regularization* effect, wherein well-trained networks naturally develop heavy-tailed singular value distributions. This phenomenon, known as Heavy-Tailed Self-Regularization, is indicative of well-formed internal representations and correlates strongly with generalization robustness and output stability.

However, when spectral diagnostics reveal deviations from these expected patterns—such as random-like spectra (under-regularization) or overly sharp eigenvalue decay (rank-collapse)—the model's internal structure becomes unstable. These spectral irregularities, though often invisible to traditional loss or benchmark-based evaluations, lead to inconsistent outputs for the same input and contribute significantly to the MVP. This is particularly critical in sentiment analysis with LLMs, where small perturbations in input or context can yield drastically different sentiment predictions.

To quantify and track these structural instabilities, Martin et al. introduce two scale-invariant metrics—the *weighted-*α and α*-Shatten norm*—which provide robust, test-data-independent indicators of a model's internal quality. These metrics not only correlate with fine-tuning effectiveness and out-of-distribution (OOD) robustness but also enable proactive identification of brittle or overfitted models before deployment.

Taken together, this spectral framework offers a powerful diagnostic lens for understanding and mitigating MVP, advancing toward a theory of interpretability grounded in random matrix theory and applicable even in the absence of labeled data.

### 3.6 Prompt sensitivity

One of the primary factors contributing to the MVP in LLM-based sentiment analysis is prompt sensitivity, where even minor variations in input phrasing can lead to significant shifts in sentiment classification. This phenomenon underscores the nondeterministic nature of LLMs, which stems from the use of stochastic decoding techniques such as temperature scaling and top-k sampling, whereas deterministic methods like beam search produce variable outputs only when explicit randomness (for example, stochastic tie-breakers) is introduced.

Although these techniques improve flexibility and adaptability, they also introduce unpredictability in sentiment predictions, making it challenging to achieve consistent and reproducible results across inference runs. Addressing prompt sensitivity requires careful prompt engineering, structured input standardization, and fine-tuning approaches to reduce variability and ensure more stable sentiment predictions.

Several studies have shown that prompt formulation significantly impacts LLM output variability, leading to inconsistent sentiment classifications in AI-driven applications. The study (Yang J. C. et al., 2024) explores how prompt variation affects voting behaviors in LLM, revealing that subtle changes in the phrasing of questions or persona framing can shift collective AI-generated decisions. This aligns with findings in sentiment analysis, where slight modifications in a prompt can cause a sentiment label to fluctuate between positive, neutral, or negative, highlighting the instability introduced by linguistic framing in LLM-based sentiment classification systems. This sensitivity not only impacts reproducibility, but also raises concerns about the reliability of AI-driven decision-making frameworks in high-risk applications such as financial analysis, legal evaluations, and policy making. As it was mentioned, recent studies confirm that LLM decision-making behavior is highly susceptible to prompt variation, further validating the claim that sentiment analysis models exhibit inconsistency depending on input wording (Loya et al., 2023).

A detailed analysis in Zhang et al. (2024) demonstrates that sentiment polarity fluctuates significantly on prompt construction, making model output highly unpredictable. Even small changes in phrasing, word emphasis, or contextual framing can lead to drastic sentiment classification changes, emphasizing the importance of structured prompt formulation. Similarly, Krugmann and Hartmann (2024) highlights that models such as GPT-3.5 and GPT-4 produce varying sentiment predictions based on prompt specificity, with explicitly structured prompts yielding greater consistency, while vague or ambiguous inputs amplify the variability of sentiment classification.

Beyond sentiment analysis, research on human-LLM interaction modes further illustrates the impact of different prompting strategies on model output. The taxonomy proposed in Gao et al. (2024) categorizes interaction techniques into standard prompting, UI-enhanced interactions, context-driven inputs and agent-facilitated prompting, each of which can contribute to variability in LLM sentiment classification. Understanding these structured interaction paradigms is essential to design consistent, robust, and reproducible sentiment analysis methodologies that reduce fluctuations in model output.

In addition, prompt sensitivity has been shown to introduce significant uncertainty in AI-driven recommendation systems. The recent study (Kweon et al., 2025) highlights that LLM-based recommendation systems face substantial volatility and uncertainty due to prompt sensitivity, variations in user history length, and stochastic inference methods, even when identical input conditions are maintained. To address this, they propose an uncertainty quantification framework that measures reliability in AI-generated recommendations and decomposes uncertainty into two key dimensions:

- Recommendation uncertainty—intrinsic ambiguity due to the complexity of the recommendation task itself.
- Prompt uncertainty—variability arising specifically from differences in prompt formulations, reinforcing the need for structured input standardization.

Prompt sensitivity is particularly critical in LLM-driven collective decision-making tasks, where LLMs act as proxies for individual opinions. Their responses can fluctuate significantly based on prompt structure and contextual framing, leading to inconsistencies in aggregated sentiment classifications. This variability is especially concerning in applications where LLMs aggregate diverse viewpoints to form a consensus, as different prompt designs may yield divergent sentiment scores, ultimately affecting overall model reliability. To minimize LLM sensitivity in sentiment classification tasks, Jarrett et al. (2025) emphasize the need for robust prompt engineering strategies and structured input standardization techniques.

### 3.7 Domain-specific challenges

MVP is further amplified in domain-specific sentiment analysis, where general-purpose LLMs struggle to adapt to specialized fields such as legal, financial, and medical domains. Domain-specific challenges arise from the inherent complexities, constraints, and specialized requirements of a particular field. These models are typically trained on broad and diverse corpora, which may not provide sufficient coverage of the nuanced vocabulary, terminology, and contextual cues specific to certain disciplines. As a result, LLMs frequently misinterpret domain-specific expressions, leading to inconsistent or unstable sentiment predictions when applied to specialized tasks. This variability underscores the need for domain-adapted training, fine-tuning methodologies, and hybrid modeling strategies to improve the accuracy, robustness, and reliability of LLMs in specialized sentiment classification tasks.

A key issue in domain-specific sentiment analysis is domain drift, where LLMs trained on general datasets fail to generalize effectively to specialized applications. As highlighted in van der Veen and Bleich (2025), LLMs exhibit greater instability when applied to finance, healthcare, and legal analysis, as their probabilistic nature often misinterpret technical language and domain-specific sentiment cues. The study argues that lexicon-based sentiment models, which rely on predefined sentiment rules, provide greater stability in structured environments where deterministic rules better capture sentiment classification nuances.

Similarly, Zhang et al. (2024) finds that LLMs trained in mixed-domain datasets exhibit higher variability when performing sentiment classification in specialized fields. The study reveals that models trained without domain-specific adaptation struggle to interpret context-dependent terminology, leading to fluctuations in sentiment classification even when presented with semantically similar inputs. This suggests that cross-domain generalization remains a persistent challenge, requiring adaptive fine-tuning, domain-specific data augmentation, and lexicon-enhanced hybrid models to stabilize LLM-based sentiment analysis.

One promising approach to mitigating domain-specific variability is the integration of hybrid models that combine lexicon-based approaches with LLM-driven sentiment classification. Using the interpretability of predefined sentiment rules with the contextual flexibility of LLMs, hybrid frameworks can achieve greater consistency and robustness across domain-specific sentiment tasks. Furthermore, techniques such as adaptive training strategies, few-shot domain adaptation, and reinforcement learning-based fine-tuning can enhance LLM performance in specialized fields by aligning model predictions with domain knowledge and linguistic conventions.

In summary, addressing domain-specific sentiment variability in LLMs requires a combination of domain-adapted training, hybrid modeling strategies, and structured fine-tuning approaches. Future research should focus on developing context-aware LLM architectures that can dynamically adjust sentiment predictions based on domain-specific linguistic cues, ensuring greater stability, accuracy, and interpretability in specialized sentiment classification tasks.

### 3.8 Reinforcement learning fine-tuning and reinforcement learning from human feedback

MVP is significantly influenced by Reinforcement Learning Fine-Tuning, which introduces shifts in post-training model predictions (Hamman et al., 2024). Reinforcement Learning from Human Feedback (RLHF) methods (Christiano et al., 2017; Ziegler et al., 2020; Ouyang et al., 2022) are widely used to align LLM behavior with human preferences, ensuring that models generate ethically sound, coherent, and contextually appropriate responses. However, while RLHF improves alignment, it also introduces new challenges in sentiment analysis, as fine-tuning adjustments can make LLM outputs less predictable, leading to inconsistencies across sentiment classifications. If alignment updates are not properly calibrated, LLMs might develop biases or unpredictable shifts in sentiment prediction over time, leading to variability in model output for identical inputs (Atil et al., 2024).

Recent research highlights how alignment-induced changes introduce unpredictability in sentiment classification. In Shorinwa et al. (2024), it is shown that RLHF models tend to shift sentiment predictions unpredictably, as the fine-tuning process modifies model behavior based on subjective human feedback, leading to inconsistent sentiment classifications across different prompts and contexts. Similarly, Beigi et al. (2024) discusses how post-training alignment mechanisms, such as safety filters, ethical constraints, and reinforcement objectives, can unintentionally distort sentiment interpretations, sometimes causing models to overcorrect or suppress certain sentiment polarities. These findings highlight a critical trade-off between model alignment and predictive stability, emphasizing the need for robust calibration techniques to maintain consistency in sentiment classification without introducing systematic distortions.

Beyond alignment challenges, fine-tuning itself introduces additional sources of model variability. Small variations in fine-tuning configurations, including random seed initialization, learning rates, training data variations, and slight modifications in hyperparameters, can lead to fine-tuning multiplicity, where multiple equally well-performing models generate conflicting sentiment classifications for the same input. This phenomenon is formalized in Hamman et al. (2024), which introduces a prediction consistency measure that demonstrates that different fine-tuned versions of the same base model can significantly diverge in the sentiment classification results due to subtle differences in training conditions. Such inconsistencies undermine the reliability of sentiment models, raising concerns about their stability, robustness, and reproducibility in high-risk applications.

### 3.9 Human-AI interaction biases and adaptation challenges

One of the key contributors to MVP in sentiment analysis is the influence of human biases during interactions with LLMs. Human users inherently interact with LLMs in a subjective way and thus can introduce inconsistencies in the results of sentiment classification. These biases arise from cognitive tendencies such as automation bias and algorithm aversion, both of which influence how users interpret and rely on AI-generated output.

- Automation bias occurs when users overtrust AI-generated sentiment assessments, accepting outputs without critical scrutiny (Parasuraman and Riley, 1997; Logg et al., 2019). This overreliance can reinforce systematic inconsistencies in model predictions, especially in ambiguous or context-sensitive sentiment classification tasks.
- In contrast, algorithm aversion occurs when users develop skepticism toward AI models after experiencing errors or unexpected sentiment output. This can lead to unpredictable interactions, where some users override model decisions even when AI-generated sentiment assessments are accurate, reducing reproducibility and stability in AI-assisted decision-making processes.

As highlighted in Passerini et al. (2025), human users exhibit distinct patterns of adaptation when engaging with LLMs, often reinforcing biased interpretations. Users who consistently trust AI-generated sentiment scores may unknowingly amplify model biases, embedding systematic distortions into the analysis pipeline. In contrast, users who frequently override AI decisions introduce variability by resisting the model output, creating instability in the consistency of sentiment assessment. This interaction-dependent bias raises concerns in critical applications, such as financial market sentiment analysis, healthcare sentiment evaluation, and policy-oriented opinion mining, where stable and unbiased sentiment predictions are essential for sound decision-making.

### 3.10 Lack of calibration in confidence scores

One of the critical factors contributing to MVP in LLM-based sentiment analysis is the lack of proper calibration of confidence scores. Confidence calibration refers to the alignment between the predicted confidence level of a model and its actual accuracy. LLMs often overestimate their confidence in incorrect predictions while underestimating it in accurate ones, leading to a disconnect between their perceived certainty and real-world performance. This miscalibration is particularly problematic in sentiment classification, where erratic confidence levels may cause inconsistent sentiment assignments, ultimately compromising the reliability of the model in decision-making processes. Without proper calibration, LLMs can misrepresent their predictive confidence, resulting in unstable sentiment scores on inference runs and reducing trust in AI-driven sentiment analysis applications.

Empirical evidence is found in Xie et al. (2025), where the discussion reflects how poorly calibrated models produce highly variable sentiment predictions, as inconsistencies in confidence estimation lead to overconfident but incorrect classifications or fluctuating sentiment scores across inference runs. Similarly, Beigi et al. (2024) highlights that LLMs often lack uncertainty-aware calibration mechanisms, emphasizing that temperature scaling, Bayesian confidence adjustments, and quantile-based methods can improve model stability in sentiment analysis.

These studies reveal that uncalibrated confidence scores introduce variability in model outputs, particularly in subjective sentiment tasks. Calibration errors lead to low trustworthiness in AI-generated sentiment classifications, which requires the integration of uncertainty quantification frameworks to improve LLM reliability in high-risk applications.

### 3.11 Evaluation metrics limitations and sentiment evaluation benchmark

One of the fundamental challenges exacerbating MVP in LLM-based sentiment analysis is the limitation of existing evaluation metrics and sentiment benchmarks. Traditional accuracy-based metrics, such as precision, recall, and the F1 score, fail to capture the variability inherent in LLM-generated sentiment classifications, as they primarily assess static performance without accounting for prediction inconsistency between inference runs. Similarly, existing sentiment benchmarks often oversimplify sentiment classification, relying on rigid categorical labels such as positive, negative, or neutral, which do not adequately reflect the complexity of real-world sentiment expressions, including sarcasm, contextual sentiment shifts, and mixed emotions. These constraints lead to inconsistent model evaluations, where the same model may yield different performance results depending on the benchmark used, further compounding uncertainty in LLM performance assessments.

Empirical evidences are found in the following two studies. In Krugmann and Hartmann (2024), the authors critique existing sentiment benchmarks, demonstrating that they often fail to capture subtle sentiment transitions and contextual dependencies, which are essential for accurate sentiment interpretation. In Ye et al. (2024), the authors highlight leaderboard discrepancies, showing that LLMs ranked highly in one evaluation setting may perform poorly in another, emphasizing the need for more robust benchmarking methods that account for sentiment stability and prediction consistency.

These findings underscore the need for improved benchmarking frameworks that incorporate uncertainty-aware metrics, stability assessments, and real-world sentiment variations to provide a more accurate evaluation of LLM performance. Standard accuracy metrics do not assess intramodel consistency, leading to fluctuating model rankings across different datasets. Uncertainty-aware evaluation frameworks that incorporate prediction confidence, sentiment stability metrics, and context-aware assessments are needed to accurately measure LLM performance.

### 3.12 The black-box nature of LLM decision-making

One of the key challenges contributing to MVP in sentiment analysis is the black-box nature of LLMs, which limits transparency and interpretability. LLMs generate sentiment classifications through complex neural architectures and large-scale statistical modeling, making it difficult to trace how and why a particular prediction is made. This opacity is problematic because identical inputs can yield different outputs, and without clear interpretability, debugging inconsistencies and mitigating variability remain significant challenges. The inability to explain these variations hinders trust in AI-driven sentiment models, particularly in high-risk applications such as finance, healthcare care and policy analysis.

A key source of interpretability challenges in LLMs is the variability introduced by the grouping mechanisms used in sentence-embedded representations. Different pooling techniques determine how token-level embeddings are aggregated into a single sentiment representation, leading to inconsistencies in sentiment classification. Mean-pooling averages token embeddings, producing stable but sometimes diluted sentiment representations by smoothing out extremes. Max-pooling, on the other hand, captures the strongest sentiment feature by selecting the highest activation per dimension, emphasizing distinct sentiment features but at the cost of higher variability in predictions. Weighted sum pooling, which dynamically adjusts token importance based on learned weights, improves classification accuracy but increases interpretability challenges, as the influence of specific tokens is difficult to trace (Zhang et al., 2024).

The pooling mechanisms directly affect the variability and interpretability of sentiment. In Xing et al. (2024), the authors show that the sentiment classification outcomes fluctuate significantly depending on the grouping method used, highlighting how subtle changes in the pooling selection can alter model predictions. Similarly, Beigi et al. (2024) underscores that the lack of interpretability in LLM intensifies the variability, making it difficult to diagnose sentiment inconsistencies. Furthermore, van der Veen and Bleich (2025) contrasts LLM-based sentiment classification with lexicon-based approaches, demonstrating that lexicon models offer greater transparency and stability by relying on explicit sentiment word mappings rather than opaque neural representations. This suggests that hybrid models integrating lexicon-based and LLM-based approaches may offer a balance between accuracy and interpretability.

A related issue is overfitting to certain sentiment patterns due to pooling biases. In multimodal sentiment tasks, for example, weighted sum grouping can misallocate importance to sentimentally neutral words, distorting the final classification. In contrast, maximum pooling can amplify noise in sentiment classification, as it over prioritizes extreme words, leading to erratic output in cases where sentiment is ambiguous. These findings highlight the need for explanation-driven pooling selection methods, ensuring that LLMs prioritize stability and interpretability alongside accuracy.

To address the black-box problem, integrating XAI techniques is essential. Methods such as SHapley Additive Explanations (SHAP) and Local Interpretable Model-Agnostic Explanations (LIME) have been applied to sentiment analysis to clarify the role of individual words in influencing sentiment classifications. Attention visualization techniques have also been used to map out which words contribute the most to sentiment decisions, offering a clearer view of how sentiment shifts occur across inference runs.

Another promising direction is the development of structured pooling calibration techniques that reduce the interpretability-accuracy trade-off. Research in Xing et al. (2024) suggests that hybrid pooling methods—combining mean and weighted sum pooling—can achieve greater consistency while preserving contextual depth, making sentiment predictions robust and interpretable. Additionally, confidence calibration strategies, such as temperature scaling and Bayesian uncertainty modeling, can help align LLM predictions with actual model confidence, improving reliability and mitigating unpredictability in sentiment classification.

In summary, the black-box nature of LLM decision-making remains a central challenge in sentiment analysis variability. However, explainability-driven techniques, optimized pooling strategies, and interpretability-aware hybrid models offer practical solutions to improve transparency and stability. By integrating these approaches, future sentiment analysis models can minimize inconsistencies, enhance trust, and ensure that LLM-based sentiment classification remains accurate and interpretable.

## 4 Analysis of the role of explainability

In this section, attention is given to XAI, a fundamental aspects for LLMs for user understanding and analysis. First, we briefly introduce XAI from a trust AI perspective in Section 4.1. In Section 4.2 we address the XAI role in LLMs. Finally, in Section 4.3 we analyze the deep structures for LLM-based XAI.

### 4.1 Explainability and trust building

XAI (Arrieta et al., 2020; Ali et al., 2023; Longo et al., 2024) can be considered an essential component of artificial intelligence. The following definition, proposed in Arrieta et al. (2020), considers the two fundamental elements when we discuss explanations: *understanding* and *audience*.

Given an audience, an *explainable AI* (XAI) is one that produces details or reasons to make its functioning clear or easy to understand.

The field of *Explainable AI* has expanded rapidly, with a wide range of technical approaches proposed to generate such explanations. The ongoing work now questions the maturity of these methods and maps the open challenges they face to support AI trust and human AI collaboration. A comprehensive conceptual reflection can be found in Herrera (2025).

The growing complexity of LLM has underscored the urgent need for effective explainability frameworks. As discussed in Herrera (2025), the shift to black-box models in recent years has raised critical concerns about transparency, interpretability, and ultimately trustworthiness of AI systems. Herrera emphasizes that the increasing reliance on advanced AI models demands a comprehensive approach to XAI, not only as a tool for clarifying internal mechanisms but also as an essential factor for fostering trust and informed human-AI interaction. This notion aligns strongly with the identified need for robust interpretability in sentiment analysis using LLMs, particularly given the high-risk and potential consequences associated with variability in sentiment output. Thus, the insights from Herrera's reflections highlight the imperative to develop contextually grounded user-oriented XAI approaches capable of demystifying AI outputs and ensuring reliable decision-making across various critical applications.

Rapid adoption and integration of LLMs across diverse sectors underscores their transformative potential. However, despite their impressive capabilities in natural language processing, these models inherently function as “*black boxes,”* obscuring the decision-making processes behind their outputs (Zhao et al., 2024). This opacity presents critical challenges, particularly concerning transparency, reliability, and ethical responsibility, that require the rigorous exploration and development of XAI methodologies.

One of the fundamental motivations for explainability in LLMs is trust building (Afroogh et al., 2024). As Luo and Specia (2024) highlight, the confidence of end-users in AI-driven systems is significantly dependent on their ability to understand the reasoning behind specific predictions or classifications. Without clear explanations, stakeholders cannot reliably calibrate model performance, leading to the potential misuse or rejection of valuable AI tools. This trust factor is especially critical in sensitive domains like healthcare, finance, and legal decision making, where misunderstood or inaccurate model predictions can have severe consequences (Zhao et al., 2024).

Furthermore, as the study (Barman et al., 2024) argues, focusing solely on transparency in the abstract may not adequately address the practical needs of diverse user groups. They emphasize the need for explainability methods that not only clarify why a model made a particular decision but also provide actionable, contextual guidance for users. Effective explainability, therefore, should move beyond mere transparency toward enabling practical, contextualized understanding that facilitates responsible AI use. This perspective suggests a shift from purely technical explanations toward pragmatic guidelines tailored to specific use cases and user proficiency levels.

### 4.2 XAI in LLMs

Addressing explainability in LLMs, Zhao et al. (2024) introduces a comprehensive taxonomy of techniques, categorizing them into local and global explanations based on their explanatory objectives. Local explanations, such as feature attribution, attention visualization and counterfactual explanations, elucidate the reasoning of the model for specific predictions, directly supporting user trust by providing concrete justifications for outputs. These techniques enable users to precisely understand which inputs or features most significantly influence individual decisions, making it possible to validate or challenge predictions on a case-by-case basis. In contrast, global explanations, which encompass approaches such as classifier investigation, mechanistic interpretability, and representation analysis, assist researchers in comprehending the overarching behaviors and structural properties of the model. These methods provide insight into internal mechanisms, hidden biases, and general knowledge encoded within the models, thus playing a crucial role in debugging, systematic model improvements, and identifying vulnerabilities or systemic issues such as embedded societal biases or the tendency of models to generate misleading information. This taxonomy emphasizes the complementary nature of local and global methods, highlighting the necessity of integrating multiple explanation types to build comprehensive, trustworthy, and interpretable LLMs.

However, current explainability methods still face significant challenges, especially due to the unprecedented scale and complexity of modern transformer-based LLMs such as GPT-4 or LLaMA. As emphasized by Luo and Specia (2024), traditional techniques such as SHAP or LIME, while valuable, often struggle with computational scalability when applied to models with billions of parameters. Moreover, existing explainability metrics frequently fall short in capturing nuances in model behaviors such as in-context learning and chain-of-thought reasoning, highlighting the need for novel, efficient, and scalable explanatory methods tailored explicitly to large-scale generative models.

In addition, explainability plays a crucial role in mitigating the ethical and social risks associated with LLM. In Zhao et al. (2024), they point out that opaque decision-making processes in models often lead to unintended biases, harmful content generation, and hallucinations, outcomes that pose substantial ethical and social implications. Robust explainability frameworks enable proactive identification and mitigation of such risks, fostering ethical alignment and responsible deployment of these powerful technologies.

In summary, advancing explainability in LLMs is essential not only to enhance user trust and model reliability but also to ensure ethical and responsible use of AI technologies in society. In the future, the development of more sophisticated, context-sensitive, and user-oriented XAI frameworks, as advocated in Barman et al. (2024), is crucial. Future research and practical guidelines should aim to bridge theoretical understandings and empirical methodologies with real-world user-centric applications, ensuring that the profound capabilities of LLMs can be channeled ethically, responsibly, and effectively.

Integrating XAI techniques into LLM-based sentiment analysis is crucial to enhance model transparency and reduce uncertainty. By providing clear insights into how models arrive at their predictions, XAI facilitates the identification and mitigation of inconsistencies and biases inherent in LLMs. Techniques such as attention mechanisms and visualization tools can highlight which parts of the input text most influence sentiment predictions, enabling users to understand and trust the model's decision-making process. For example, employing attention-based explanation methods can reveal how specific words or phrases contribute to the overall sentiment classification, thereby offering a more interpretable and reliable analysis. Furthermore, approaches grounded in linguistic theory, such as construction grammar, can further clarify how LLMs internalize complex linguistic patterns and meanings, thus providing deeper explanatory insights into model behavior and potential misunderstandings (Weissweiler et al., 2023). By adopting these combined XAI strategies, stakeholders can achieve a deeper understanding of model behavior, leading to more consistent and trustworthy sentiment analysis results (Mabokela et al., 2024; Weissweiler et al., 2023).

### 4.3 Deep structures for LLM-based XAI

As highlighted by Da et al. (2025), the reasoning processes in LLMs are not always stable, with different paths leading to the same or different final conclusions depending on how the model structures its logical dependencies. This structural uncertainty, when applied to sentiment analysis, can result in divergent sentiment scores for the same input. Addressing this challenge requires adopting structured methods for explanation-based uncertainty quantification, such as reasoning topology modeling, to systematically capture and mitigate variability in sentiment interpretation.

Explainability in LLMs is not only about generating user-friendly rationales but also about uncovering the deeper structures and statistical signatures that govern model behavior. A key contribution to this broader vision comes from Martin et al. (2021) and Martin and Mahoney (2021), who propose a diagnostic approach that bypasses traditional benchmarking. Their framework introduces spectral metrics—such as heavy-tailed power-law exponents and Shatten norms—to assess model generalization and robustness without relying on labeled test data. These insights are vital in dynamic or high-risk domains, such as finance or healthcare, where labeled validation sets are unavailable or infeasible. Spectral irregularities in weight matrices signal deviations from the expected implicit self-regularization behavior of well-trained models, offering a scalable and quantitative means of identifying brittle or overfitted LLMs. By exposing these internal statistical pathologies, this line of work provides a structural lens for assessing the trustworthiness of black-box models and directly addresses MVP rooted in hidden instabilities.

At the same time, recent advancements in mechanistic interpretability (MI)—as surveyed by Rai et al. (2025) and operationalized by Anthropic (Ameisen et al., 2025; Lindsey et al., 2025)—focus on reverse-engineering internal circuits and causal pathways in LLMs. These bottom-up methods trace how models arrive at decisions by isolating conceptual features, circuits, and attribution graphs, moving explainability beyond surface rationales toward faithful, structure-aware analysis. While spectral diagnostics provide a global, model-level signal of risk, MI techniques like activation patching and circuit tracing reveal the fine-grained reasoning structures within models, enabling developers to audit behaviors such as planning, hallucination, or misalignment.

Building on this foundation, García-Carrasco et al. (2024) and García-Carrasco et al. (2025) introduce a complementary strategy that further operationalizes mechanistic interpretability by extracting interpretable, task-specific circuits from LLMs. Their work demonstrates that it is possible to isolate compact subnetworks responsible for specific behaviors—such as acronym prediction or sentiment detection—without retraining the model. This modular interpretability not only enhances our ability to trace causal pathways within the model but also significantly reduces inference costs by pruning irrelevant components, yielding models that are both faster and more transparent. Crucially, this approach bridges the global-local interpretability divide: by first identifying global model regions responsible for a task and then drilling down to functional circuits, it supports stakeholder-specific views—regulators, developers, and end-users alike—on model behavior. In the broader interpretability landscape, these circuit extraction techniques serve as mid-level interfaces that connect model-wide spectral diagnostics with neuron-level interpretability, effectively scaffolding a full-stack explainability architecture.

Together, these complementary approaches—statistical and mechanistic—create a unified interpretability framework. This integration enables scalable evaluation (via spectral diagnostics) and localized causal validation (via circuit tracing), forming the foundation for transparent, accountable, and safe deployment of LLMs. As emphasized by Amodei (2025), such frameworks must evolve from ad hoc analysis tools into essential infrastructure—capable of detecting alignment risks, legal noncompliance, and model deception—before frontier models are widely adopted in governance-critical and safety-sensitive contexts.

***Dimension frontiers***. In summary, the exploration of explainability plays a vital role in addressing the uncertainty and variability inherent in LLM-based sentiment analysis. Given that LLM sentiment predictions are particularly susceptible to variability driven by stochastic inference, prompt sensitivity, and training biases, it is crucial to incorporate robust and interpretable explanation methods. Effective explainability not only provides clarity on how and why sentiment classifications vary, but also establishes trust and facilitates user validation of AI-generated sentiments.

As a notable recent contribution within this frontier, Nguyen et al. (2024) introduce the novel Sentiment Reasoning task in healthcare care, demonstrating that rationale-augmented sentiment classification significantly improves both interpretability and performance, an approach that directly supports our goal of improving model transparency and reliability in high-stakes domains impacted by the MVP.

Aligning comprehensive XAI frameworks with methods for uncertainty quantification and variability mitigation forms an essential foundation for improving the stability, reliability, and trustworthiness of LLM-based sentiment analysis systems, directly addressing the central themes of this perspective study.

## 5 Challenges for model variability problem in LLM-based sentiment analysis

The evaluation of LLM-based sentiment analysis remains deeply affected by the MVP, which undermines the consistency, interpretability, and trustworthiness of model predictions across different domains and use cases. In Section 3, we outline a conceptual foundation by identifying a dozen fundamental reasons for MVP. These underlying sources of variability provide a theoretical lens through which the behavior of LLMs can be better understood and scrutinized.

Building on that foundation, this section presents fourteen key challenges that operationalize these theoretical insights into specific problem areas within LLM-based sentiment analysis. Each challenge is drawn from a synthesis of current literature and corresponds directly to one or more of the previously identified root causes. In doing so, we establish a clear analytical bridge between high-level variability factors and actionable avenues for mitigation. These challenges not only frame the practical impact of MVP, but also highlight solution spaces, ranging from spectral diagnostics to explainability frameworks, that may offer paths toward reducing variability and improving the robustness of LLM-based sentiment analysis systems.

### 5.1 Lack of standardized and stability-aware benchmarking frameworks

The evaluation of sentiment analysis models is based primarily on traditional performance metrics such as precision, accuracy, recall, and the F1 score. However, these metrics are insufficient to assess variability in LLM-based sentiment analysis, as they do not account for fluctuations in model output over multiple inference runs. Although standard sentiment benchmarks exist, they often fail to measure stability and consistency, making it difficult to determine whether an LLM can reliably classify sentiment in different contexts and prompts.

One of the key issues is that models that rank in one benchmark may perform poorly in another, as highlighted in Ye et al. (2024), demonstrating benchmarking inconsistencies in sentiment analysis. Furthermore, traditional evaluation metrics do not consider how sentiment classification changes over time due to model updates, prompt variations, or data set changes. This gap in the evaluation leads to a misleading perception of the reliability of the model, which prevents a proper assessment of the stability of the prediction of sentiments.

The instability of sentiment classification outputs in LLMs is exacerbated by the model's sensitivity to prompt construction and hyper-parameter settings. Recent research demonstrates that sentiment scores can vary significantly due to minor prompt re-wording or changes in decoding parameters, reinforcing the need for methods to improve stability and reliability in sentiment classification (Loya et al., 2023).

#### 5.1.1 Potential solution

A potential solution is to introduce stability-aware benchmarking frameworks that incorporate uncertainty-aware evaluation metrics, such as confidence scores based on entropy and stability indices—the former is obtained by running the model several times on the same input, averaging the predicted class-probability vectors, and treating lower entropy as higher confidence, while the latter measures the percentage of repeated runs that agree with the most frequent label, thereby quantifying run-to-run repeatability (Ye et al., 2024; Hamman et al., 2024). Furthermore, cross-benchmark validation using multiple domain-specific datasets could improve sentiment-classification robustness, ensuring that evaluations reflect real-world performance variations rather than over-fitting to specific benchmarks.

### 5.2 Sensitivity to prompt variability and input reframing

LLMs are highly sensitive to variations in prompt phrasing, which means that small changes in input wording can lead to significantly different sentiment predictions. This issue makes it difficult to replicate the results of sentiment classification consistently, reducing trust in LLM-based sentiment analysis applications.

Studies such as Zhang et al. (2024) and Krugmann and Hartmann (2024) reveal that GPT-3.5 and GPT-4 generate different sentiment scores based on prompt specificity, even when the sentiment meaning remains unchanged. Furthermore, the lack of standardized prompt design guidelines makes it difficult for researchers to evaluate whether the sentiment output of a model is a result of genuine contextual understanding or a by-product of prompt sensitivity.

Recent work by Reveilhac and Morselli (2024) shows that LLMs such as ChatGPT exhibit notable shifts in decision-making behavior based on linguistic, cultural, and contextual cues embedded within the prompts. The study highlights how model outputs fluctuate depending on the ideological framing of political prompts and the language in which queries are presented. This variability aligns with our findings that sentiment analysis outputs are highly prompt-sensitive, requiring standardized prompt engineering strategies to mitigate inconsistencies.

#### 5.2.1 Potential solution

Given the substantial impact of prompt sensitivity on the variability of sentiment analysis, it is essential to develop systematic techniques to mitigate its effects. Key strategies include:

- Standardization of prompt design to reduce linguistic variability and improve consistency in model responses.
- Implement prompt optimization frameworks that ensure that LLMs generate sentiment predictions with minimal deviation across inference runs.
- Integrating uncertainty-aware modeling to detect when model predictions are likely to fluctuate due to prompt variations.
- Using ensemble-based sentiment evaluation methods, where multiple prompt structures are tested to derive more robust and consensus-driven sentiment scores.

By addressing prompt sensitivity through structured input standardization and explainability-driven interventions, LLM-based sentiment analysis can achieve greater stability, reliability, and reproducibility, ensuring its effective deployment in high-risk AI-driven decision-making environments.

### 5.3 Epistemic and aleatoric uncertainty in the interpretability of the model

LLMs suffer from two major types of uncertainty: epistemic uncertainty, which arises from knowledge limitations within the model, and aleatoric uncertainty, which is caused by inherent noise and ambiguity in training data. These uncertainties make it difficult for LLMs to produce stable sentiment classifications, leading to inconsistencies in sentiment predictions.

As discussed in Shorinwa et al. (2024), epistemic uncertainty is the result of incomplete training data, causing LLMs to misinterpret sentiment in unseen or ambiguous contexts. Furthermore, aleatoric uncertainty, as highlighted in Beigi et al. (2024), arises due to human annotation errors, slang, sarcasm, and domain shifts, leading to inconsistent sentiment classifications. This dual source uncertainty problem reduces the reliability of sentiment analysis models. In Reveilhac and Morselli (2024), the authors emphasize how epistemic uncertainty affects decision making in LLM-powered voting systems, and Passerini et al. (2025) explores how mutual adaptation between humans and LLM can reduce or amplify epistemic uncertainty.

The association of aleatoric and epistemic uncertainty with the studies analyzed reveals that both sources of uncertainty are the main contributors to LLM variability in sentiment analysis. Aleatoric uncertainty arises from intrinsic linguistic ambiguities, while epistemic uncertainty arises from knowledge limitations within the model itself.

#### 5.3.1 Potential solution

Effective mitigation requires a combination of context-aware sentiment embeddings, structured fine-tuning methodologies, confidence calibration frameworks and explainability-driven feedback mechanisms. By addressing these uncertainties, we can significantly improve the robustness, reliability, and interpretability of sentiment classification in LLM-based systems.

To mitigate uncertainty in sentiment analysis, models should be trained with uncertainty-aware learning techniques, such as Bayesian deep learning or Monte Carlo dropout, which quantify and account for confidence levels in sentiment predictions. Additionally, incorporating explainability methods (e.g., attention visualization) can help researchers diagnose whether sentiment shifts are due to knowledge gaps or data-driven noise, improving interpretability and reliability.

### 5.4 Diagnosing and mitigating model variability through structural and mechanistic interpretability—without ground truth

One of the central challenges in deploying LLMs is assessing and controlling model variability in settings where labeled test data is scarce or unavailable—a common scenario in high-risk domains such as finance, healthcare, and legal analytics. This MVP is exacerbated by the opaque black-box nature of LLMs, their stochastic inference processes, sensitivity to prompt phrasing, and susceptibility to training data biases. Traditional evaluation techniques fall short under such constraints, calling for alternative methods that offer reliable diagnostics without requiring access to ground truth.

To address this, Martin et al. (2021) and Martin and Mahoney (2021) propose a spectral diagnostic framework that evaluates the quality of the model through the intrinsic statistical properties of the weight matrices. Their analysis shows that well-generalizing models exhibit Heavy-Tailed Self-Regularization—a spectral pattern detectable through power-law exponents (e.g., α-Shatten norms). When these spectral signatures deviate from expected ranges (e.g., α > 2.3), it signals potential instability, under-regularization, or overfitting. These indicators can be monitored post hoc or during training to assess model robustness, making them especially useful in environments where traditional performance metrics are inaccessible. Integrating these diagnostics into practice enables checkpoint selection, spectrum-aware fine-tuning, and reproducible benchmarking in LLM-based sentiment analysis.

In parallel, recent advances in mechanistic interpretability—such as circuit tracing, attribution graphs, and modular subnetwork extraction—provide complementary tools to uncover causal structures and internal reasoning paths within transformer-based models (Rai et al., 2025; García-Carrasco et al., 2024, 2025; Ameisen et al., 2025).

While spectral diagnostics offer a global perspective on model stability, mechanistic methods enable localized analysis of prediction pathways, facilitating a deeper understanding of why and how variability arises in model outputs. Together, these approaches offer a multilayered diagnostic framework—combining global spectral insights with fine-grained causal tracing—to reliably assess and mitigate model variability in LLMs, even in the absence of labeled ground truth.

#### 5.4.1 Potential solution

Fusing spectral and mechanistic approaches offers a unified interpretability framework capable of diagnosing and mitigating LLM variability without the need for labeled validation data. In practice, tools like WeightWatcher can be used to monitor spectral health during training or deployment, while mechanistic techniques (e.g., circuit extraction or attribution analysis) help verify reasoning consistency in specific tasks such as sentiment classification. This combination enables proactive detection of failure modes, structural risk, and misaligned reasoning in real-time, thereby enhancing trust, reproducibility, and stability in LLM-based applications.

### 5.5 RLHF-induced variability

RLHF and fine-tuning methodologies play a crucial role in aligning LLM sentiment predictions, but they also introduce new sources of variability that must be carefully managed. Future research should focus on developing structured RLHF calibration frameworks, stability-aware fine-tuning techniques, and ensemble-based evaluation strategies to ensure consistency in LLM-based sentiment classification tasks. By addressing these alignment-induced inconsistencies, sentiment analysis models can achieve greater reliability, reproducibility, and robustness, enabling more effective deployment in real-world decision-making environments.

#### 5.5.1 Potential solution

Addressing the variability introduced by RLHF and the fine-tuning multiplicity requires the implementation of structured calibration techniques and stability-aware optimization frameworks. Key strategies include:

- Confidence-aware RLHF adjustments: fine-tuning alignment strategies should incorporate confidence estimation techniques that assess the impact of reinforcement learning updates on prediction consistency before deployment.
- Fine-tuning stability protocols: employing stability-driven retraining techniques, such as ensemble fine-tuning, iterative feedback alignment, and controlled learning rate decay, can reduce fluctuations in sentiment classification.
- Ensemble-based consistency evaluation: using multiple fine-tuned model checkpoints and aggregating predictions through voting mechanisms can increase robustness and reduce the influence of alignment-induced shifts.
- Uncertainty quantification techniques: implementing quantile-based calibration, epistemic uncertainty estimation, and Monte Carlo dropout methods can help quantify variability in fine-tuned sentiment models, ensuring more reliable output.

### 5.6 Sensitivity to model updates and fine-tuning variability

One of the major challenges in ensuring stability in sentiment analysis is the variability introduced by iterative model updates and fine-tuning strategies. Fine-tuned LLMs, even when trained on similar datasets with minor modifications, may produce contradictory sentiment predictions for identical inputs, introducing inconsistencies in high-risk applications like finance, healthcare, or customer feedback analysis. This issue is exacerbated by the need for frequent model retraining due to data drift, evolving user language, and regulatory constraints such as GDPR's “right to be forgotten,” which requires data removal and potential model retraining.

Recent work by Hamman et al. (2024) systematically examines the fine-tuning multiplicity, demonstrating that models fine-tuned under slightly different conditions (e.g. random seed initialization, additional training samples) can exhibit arbitrary sentiment classification results. Their proposed prediction consistency metric quantifies a model's susceptibility to fine-tuning variability and offers a probabilistic measure of prediction robustness. Addressing this challenge requires developing stability-sensitive fine-tuning protocols, uncertainty-sensitive retraining strategies, and adaptive sentiment calibration techniques that mitigate inconsistencies arising from iterative model updates.

#### 5.6.1 Potential solution

Addressing the instability caused by the variability in fine-tuning requires a combination of stability-aware training protocols, such as variance-penalizing or checkpoint-agreement losses (Hamman et al., 2024; Xie et al., 2025), uncertainty-quantification techniques (e.g., predictive-entropy or Monte-Carlo-dropout intervals that trigger selective retraining (Ye et al., 2024; Kweon et al., 2025)), and adaptive calibration strategies, notably temperature scaling or isotonic regression applied after each fine-tuning cycle to keep class probabilities well-calibrated (Beigi et al., 2024). Stability-aware training protocols, uncertainty quantification techniques, and adaptive calibration strategies. One approach is to implement ensemble fine-tuning, where multiple instances of a fine-tuned model are trained with different initializations and hyperparameters, and their outputs are aggregated using consensus mechanisms to enhance prediction robustness. In addition, regularization techniques, such as variance penalization during training, can help reduce divergence among fine-tuned models. Another promising method is progressive fine-tuning, where model updates are applied in smaller, controlled increments to minimize abrupt shifts in sentiment classification behavior. Finally, continuous learning strategies that incorporate past training checkpoints while adapting to new data distributions can improve the consistency of model update, reducing the likelihood of erratic sentiment classification shifts over time.

### 5.7 Reproducibility and stability in sentiment analysis

One of the biggest challenges in the deployment of LLMs for sentiment analysis is ensuring reproducibility and stability. The lack of deterministic behavior in LLM introduces output fluctuations that affect reliability in real-world decision-making applications. Stability and reproducibility remain key unresolved challenges in LLM-based sentiment analysis.

As highlighted in Atil et al. (2024), LLMs often generate inconsistent responses even when the same input is provided under supposedly deterministic settings (e.g., temperature = 0, fixed seeds, identical prompts). This behavior significantly affects use cases that demand repeatability, such as financial sentiment analysis or healthcare AI applications. This unpredictability arises from temperature variations, randomness in the decoding, and latent model instabilities, which can cause the same sentiment query to be interpreted differently between runs. The study further notes that longer output sequences correlate negatively with stability, meaning that models generating verbose explanations tend to exhibit even greater variability.

#### 5.7.1 Potential solution

To address this challenge, it is crucial to establish new standardized benchmarking protocols that assess model consistency over repeated runs. In addition, integrating ensemble techniques or model voting mechanisms could improve decision stability by averaging individual model fluctuations. Furthermore, fine-tuning strategies that explicitly optimize for deterministic behavior and constrain variance using regularization techniques could mitigate these inconsistencies, fostering a more reliable foundation for LLM-driven sentiment analysis.

On the other hand, regarding inference stochasticity in general, and the temperature variations in particular, we can pay attention to different actions. Among these to mitigate the temperature-induced variability, several strategies can be considered:

- Use of low-temperature sampling (*T*≈0): For tasks requiring deterministic or audit-ready outputs, setting the temperature to near zero can reduce stochasticity. However, this may also increase the exposure to alignment flaws or high-confidence errors.
- Multi-sample aggregation: Sampling multiple outputs at varied temperatures and applying majority-vote or confidence-based aggregation can smooth stochastic spikes.
- Temperature calibration curves: Tracking output divergence across a range of temperature settings allows the identification of robust operating zones for a given task or domain.

### 5.8 Human-LLM feedback loop and confirmation bias

Another significant challenge in LLM-based sentiment analysis is the reinforcement of feedback loops that arise from repeated human-LLM interactions. This issue stems from confirmation bias, where users unintentionally reinforce preexisting beliefs by influencing how LLMs generate responses. Rather than acting as neutral sentiment classifiers, models can gradually align with user expectations, amplifying subjective biases rather than maintaining objective sentiment analysis.

In Passerini et al. (2025), researchers discuss how mutual adaptation between humans and LLMs can either enhance decision-making synergy or exacerbate cognitive biases. In sentiment analysis tasks, this manifests itself when users consistently interact with an LLM in a way that skews its responses toward a specific sentiment polarity. Over time, models may reinforce the user's preferred sentiment interpretations, leading to skewed sentiment scores that lack objective grounding. This self-reinforcing loop compromises neutrality, particularly in sensitive applications such as public opinion analysis, market research, and policy evaluation. Another significant challenge is the way human feedback loops influence LLM behavior, leading to reinforcement of biased sentiment interpretations.

Human-LLM interaction can lead to a self-reinforcing feedback loop, where model responses adapt to user expectations rather than maintaining an objective stance. As users engage with sentiment analysis systems over time, the likelihood of confirmation bias increases, where the model prioritizes responses that align with previously accepted sentiment patterns, rather than evaluating text based on an independent, neutral linguistic framework. This issue is exacerbated by the iterative fine-tuning of LLMs based on user interactions, as models continuously learn from their own outputs and user preferences, further embedding biases into future predictions.

#### 5.8.1 Potential solution

To mitigate this problem, adaptive bias correction techniques and trust-based LLM calibration are essential. Possible solutions include:

- Counter-bias mechanisms, where models periodically introduce neutral or opposing perspectives to break reinforcing feedback cycles.
- Diversity-driven sentiment prompts, encouraging users to interact with varied perspectives, preventing model drift toward biased sentiment outputs.
- Interactive explainability features, allowing users to assess the reasoning behind sentiment predictions, thereby fostering more balanced decision-making.
- Adaptive AI-human collaboration frameworks, where LLMs dynamically adjust response strategies based on user interaction patterns, ensuring more stable and objective sentiment analysis outputs.

By integrating bias-aware interaction models, human-AI collaboration can be optimized, ensuring higher reliability, objectivity, and consistency in sentiment classification.

### 5.9 Bias-induced variability and domain adaptation issues

LLMs inherit biases from their training data, leading to systematic sentiment variations when analyzing content from different demographics, industries, or social groups. This bias-induced variability can cause sentiment misclassification, making it difficult for models to maintain consistent results across different domains.

A major concern in LLM-based sentiment analysis is bias and inconsistency in sentiment aggregation, where models generate contradictory sentiment scores for the same input text based on prompt structure, response aggregation methods, or temperature settings. Research on LLM-based voting mechanisms suggests that aggregation inconsistencies are particularly pronounced in multiwinner voting settings, where the ordering of options and voting methodologies affects decision-making outputs (Yang J. C. et al., 2024). These findings indicate that similar aggregation challenges exist in sentiment analysis tasks, where ranking-based sentiment scoring vs. binary sentiment classification can produce conflicting results. This highlights the importance of structured sentiment evaluation strategies, where results are normalized across prompts and calibrated to mitigate sensitivity to order effects.

As shown in Krugmann and Hartmann (2024), GPT-3.5 exhibits positive sentiment bias, while GPT-4 leans more neutral or negative, demonstrating how sentiment polarity can vary between model versions. Furthermore, van der Veen and Bleich (2025) highlights that LLMs struggle with domain-specific sentiment tasks, making them unstable when applied to specialized fields like finance or healthcare. These findings emphasize that bias-related variability affects the reproducibility of sentiment classification.

As demonstrated by Reveilhac and Morselli (2024), differences in the versions of the LLM model can lead to inconsistent decision-making outcomes, even for politically relevant tasks such as voting simulation. The study found that the ideological positioning of ChatGPT changes between GPT-3.5 and GPT-4, with version-dependent biases that affect the stance taken on issues of direct democracy. In sentiment analysis, such model drift raises concerns about reproducibility, making longitudinal stability evaluations essential for benchmarking consistency in AI-generated sentiment predictions.

#### 5.9.1 Potential solution

A key strategy to mitigate bias-induced variability is to incorporate fairness-sensitive training methods, such as bias-correction techniques [temperature scaling, isotonic regression and class-balanced re-weighting, each of which re-calibrates output probabilities toward the empirical class distribution (Beigi et al., 2024)] and adversarial debiasing methods that attach a gradient-reversal discriminator so the encoder learns attribute-invariant representations, reducing version-induced drift (Shorinwa et al., 2024). Additionally, fine-tuning LLMs on domain-specific sentiment data can improve stability and prevent misclassification when the models are applied to specialized contexts.

### 5.10 Achieving consensus through ensemble approaches among LLM in sentiment analysis

One promising approach to mitigating MVP in sentiment analysis involves leveraging ensemble methods, where multiple LLMs independently evaluate the same textual input, subsequently seeking a consensus or aggregated decision. Two studies (Agrawal et al., 2024; Abburi et al., 2023) demonstrate that combining multiple models can enhance robustness and predictive stability. However, effectively achieving consensus among independently operating LLMs for sentiment classification remains challenging, as individual models may differ significantly in their predictions due to inherent architectural differences, different training procedures, and varied inference strategies.

A primary issue is managing divergent outputs from multiple models, as ensemble members can provide contrasting sentiment scores due to differences in training data, biases, prompt sensitivity, and stochasticity in token generation. In Agrawal et al. (2024), it is highlighted that ensembles can sometimes amplify rather than reduce variance if not appropriately managed, particularly when member models differ substantially in reliability or calibration.

In Abburi et al. (2023), additional complexities are emphasized in aligning and interpreting the probabilistic predictions generated by each member of the ensemble, creating difficulties in determining a classification of unified feelings. Without a structured mechanism for aggregation and reconciliation, ensemble approaches risk exacerbating interpretability challenges, increasing computational costs, and potentially diminishing user trust.

A significant challenge in sentiment analysis is to ensure consistency when aggregating the outputs of multiple LLMs to derive a final sentiment classification. This issue becomes particularly relevant in LLM-based ensemble voting mechanisms, where multiple models contribute to a collective decision. In Jarrett et al. (2025), the authors explore how digital representatives can effectively simulate human decision making within collective settings, providing insights into how LLMs can be aligned for more structured decision aggregation. However, as our study highlights, variability in individual LLM predictions complicates the consensus-building process, as slight variations in model outputs can lead to significantly different aggregated sentiment classifications. Addressing this challenge requires the integration of consensus-driven voting algorithms, adaptive weighting schemes, and trust-based model selection mechanisms to enhance consistency across multi-LLM systems.

#### 5.10.1 Potential solution

To address these challenges, a structured and adaptive ensemble consensus strategy can be adopted. Specifically, methods such as weighted aggregation, confidence-based voting, or adaptive majority voting informed by uncertainty quantification metrics can help effectively reconcile divergent sentiment scores into a unified and trustworthy classification. Inspired by Agrawal et al. (2024), incorporating token-level weighting or boosting mechanisms can further enhance consensus stability. Furthermore, the integration of transparent and explainable aggregation mechanisms, such as visualization tools that explicitly illustrate how each model contributes to the final sentiment decision, would significantly improve interpretability and user trust. Such consensus-driven ensemble methods not only promise more stable and reliable sentiment predictions but also foster greater acceptance and confidence in LLM-based sentiment analysis outcomes.

### 5.11 Applying knowledge distillation to mitigate MVP in LLMs for sentiment analysis

LLMs have significantly advanced natural language processing tasks, including sentiment analysis. However, their substantial computational requirements and inherent model variability pose challenges for practical deployment. Knowledge distillation (Gu et al., 2024a,b)—a technique in which a smaller “student” model learns from a larger “teacher” model— offers a potential solution to these issues. The primary objective is to maintain the performance of the original model while reducing its size and computational demands. However, effective application of knowledge distillation to LLMs, particularly in the context of sentiment analysis, presents specific challenges that need to be addressed.

Training Smaller Language Models (SLM) through knowledge distillation presents several challenges. Firstly, LLMs possess intricate architectures capable of capturing nuanced language patterns, making it difficult to transfer this sophisticated knowledge to smaller models without significant performance loss. Additionally, standard distillation methods may not be optimal for generative language models due to differences in output distributions between teacher and student models, leading to suboptimal performance in the distilled models. Moreover, LLMs often produce uncertain or ambiguous outputs, especially in tasks like sentiment analysis that involve nuanced expressions; effectively capturing and transferring this uncertainty during distillation is complex, but crucial for maintaining model reliability. Recent studies have explored methods to improve knowledge distillation for small language models, aiming to address these challenges and improve the efficiency and effectiveness of the distillation process (Yam and Paek, 2024).

#### 5.11.1 Potential solution

To enhance the performance and efficiency of SLM, researchers have explored the integration of knowledge distillation and fine-tuning techniques. For example, the MiniPLM framework (Gu et al., 2024b) refines the distribution of training data using the teacher's insight, enabling the student model to achieve competitive performance with reduced complexity. Additionally, fine-tuning the distilled model on task-specific data allows it to adapt to particular nuances, further enhancing its effectiveness in applications like sentiment analysis. This combined approach not only maintains robust performance but also ensures that smaller models are more adaptable and efficient in real-world scenarios.

### 5.12 Ensuring consistency and robustness in enhanced sentiment analysis crowd decision making through prompt-based LLM

A significant challenge in leveraging LLMs for crowd decision-making involves ensuring consistency and robustness in sentiment classification outputs derived from structured prompt interactions. Despite the flexibility and effectiveness of prompt design strategies with models such as ChatGPT, these approaches are highly susceptible to variability, as small modifications in prompt wording, context framing, or inference parameters can result in substantial fluctuations in sentiment scores or classifications. The problem is exacerbated when opinions aggregated from texts from various users are sensitive to variations in language nuances, leading to uncertain or conflicting decisions (Herrera-Poyatos et al., 2025).

Key issues associated with this challenge include the inherent sensitivity of LLMs to slight prompt modifications, causing unpredictable shifts in sentiment polarity assessments. As highlighted in Herrera-Poyatos et al. (2025), this sensitivity leads to inconsistencies that hinder stable decision-making processes, undermining the reliability of ESA-CDM systems. Furthermore, the complexity of aggregating multiple sentiment evaluations from various crowd sources magnifies these inconsistencies, complicating consensus formation, and potentially reducing trustworthiness. Furthermore, without adequate transparency and explainability mechanisms, stakeholders cannot readily identify the rationale behind divergent model outcomes, intensifying the uncertainty surrounding the final decisions.

#### 5.12.1 Potential solution

To develop structured frameworks and standardize and optimize prompt designs, minimizing variability through well-defined linguistic templates. Additionally, employing ensemble consensus methods, as suggested in recent literature, can significantly stabilize LLM-based sentiment predictions by aggregating outputs from multiple structured prompts or diverse LLM instances, smoothing out variations and enhancing robustness. Integrating uncertainty quantification metrics into the CDM workflow could further help assess the reliability of aggregated sentiment scores, allowing decision makers to explicitly evaluate confidence levels. Finally, coupling these methodological strategies with explainability tools, such as attention visualization or token attribution methods, would increase the transparency of decision-making processes, facilitating trust and acceptance among stakeholders in ESA-CDM contexts utilizing LLM.

### 5.13 Model variability in the era of open-source LLM proliferation

Rapid adoption of open-weight models such as DeepSeek-R1 has fundamentally shifted the landscape of LLM research and deployment. No longer lagging behind their proprietary counterparts, open-source LLMs now offer state-of-the-art reasoning and instruction-following abilities, with transparent architectures and permissive licensing. These models are increasingly chosen for cloud integration, mass-market applications (e.g., Perplexity), and downstream fine-tuning. However, this shift introduces new and urgent challenges in terms of model variability, reproducibility, and trustworthiness.

Unlike proprietary models that are version-locked and centrally maintained, open-source models such as DeepSeek-R1 and Falcon are being adapted, compressed, and fine-tuned in thousands of independent forks. This leads to substantial behavioral drift, including variation in output under prompt rephrasing, inconsistent refusal behavior, and degraded factuality due to uneven fine-tuning practices. Compression techniques such as CompactifAI (Tomut et al., 2024), although valuable for scalability, can amplify these variabilities unless systematically controlled.

This fragmentation makes it difficult for researchers and enterprise users to establish behavioral guarantees or audit model decisions. Addressing the MVP in open-source ecosystems is critical for a trustworthy deployment. This challenge requires the development of evaluation protocols and mitigation strategies to ensure stability, safety, and consistency, even across diverse implementations, compression states, and usage contexts. As open models begin to replace closed APIs in production systems, this issue will become increasingly central to the future of reliable open LLMs.

#### 5.13.1 Potential solution

Addressing model variability in the context of open source LLM proliferation requires a multifaceted strategy that combines methodological standardization, ensemble stabilization, and post-deployment monitoring. One promising direction is the development of benchmarked prompt engineering templates and shared inference protocols that reduce behavioral drift between implementations. In parallel, ensemble-based approaches, such as majority voting or confidence-weighted aggregation across multiple fine-tuned instances, can improve stability by smoothing out individual model fluctuations. Additionally, incorporating calibration and consistency checking techniques, such as response entropy tracking and top k divergence metrics, can help identify and correct unstable behaviors. It is also crucial to adopt reproducibility-aware practices, including model cards and traceable configuration logs, to document the provenance and training variations of open source forks. Lastly, open models should integrate lightweight explainability modules or behavioral validation tests (e.g., prompt response unit tests) during compression or fine-tuning, to proactively detect and mitigate variability. These strategies aim to preserve the flexibility of open source LLMs while introducing essential reliability layers for production- and research-grade applications.

### 5.14 Lack of explainability and trustworthiness in the output of sentimental polarity

As we have discussed in Section 4, one of the central challenges in the analysis of sentiment with LLMs is their lack of transparency and explainability, which undermines trustworthiness and reliability. LLMs operate primarily as black-box systems, obscuring the reasoning behind sentiment polarity predictions and making it challenging for users to understand how conclusions are reached. Unlike lexicon-based methods, where explicit word-sentiment mappings allow transparent reasoning, LLMs rely on intricate neural network representations. This complexity impedes the ability of users and stakeholders, especially in sensitive applications, to trust sentiment predictions, as decisions based on unclear or inconsistent outputs pose substantial risks.

The opaque nature of LLMs creates significant hurdles for reproducibility and trustworthiness. Users cannot reliably interpret why particular sentiment scores are assigned, especially when these scores fluctuate over multiple inference runs, exacerbating the MVP. This lack of interpretability not only compromises the transparency of the model, but also undermines trust, as stakeholders cannot confidently understand or justify sentiment predictions in critical scenarios. Research such as van der Veen and Bleich (2025) explicitly contrasts the interpretability benefits of lexicon-based approaches with the inherent unpredictability and lack of transparency of LLM-based sentiment analysis. Furthermore, as shown in Beigi et al. (2024), LLM sentiment scores frequently fluctuate with minor changes in model alignment methods or training data nuances, highlighting how inherent black-box characteristics amplify concerns about model trustworthiness and reliability.

One of the key challenges in ensuring the interpretability of LLM-based sentiment analysis is understanding how different interaction paradigms influence sentiment classification decisions. The taxonomy presented in Gao et al. (2024) categorizes the interaction modes that structure human-LLM exchanges, providing insights into how structured prompting, UI-enhanced reasoning, and agent-facilitated collaboration affect model transparency. These structured approaches can improve explainability by making LLM-generated sentiment scores more interpretable, reducing user uncertainty, and fostering greater trust in AI-driven sentiment analysis. In Da et al. (2025), the study introduces a reasoning topology framework that decomposes LLM-generated explanations into structured components, allowing for a more precise evaluation of uncertainty. This approach is particularly relevant for sentiment analysis, where model variability can often arise from subtle differences in justification paths. Using structured uncertainty quantification, models can be designed to provide more consistent and interpretable sentiment predictions, reducing the overall impact of stochastic variability.

#### 5.14.1 Potential solution

To improve interpretability, reliability, and trustworthiness in sentiment analysis, the integration of XAI techniques is crucial. Methods such as SHAP (SHapley Additive Explanations) and LIME (Local Interpretable Model-Agnostic Explanations) can be utilized to visualize and quantify the contributions of input tokens, enabling a clearer tracing of sentiment decisions. Another effective approach involves creating hybrid sentiment analysis systems that integrate LLM's contextual awareness with transparent, lexicon-based sentiment rules. Furthermore, structured uncertainty quantification and calibration frameworks, such as confidence-based prediction intervals or ensemble models, can further enhance reliability, ensuring that users not only receive interpretable outputs but also consistently reliable sentiment classifications. By explicitly addressing interpretability and trustworthiness alongside reliability, sentiment analysis models become better aligned with stakeholder expectations, fostering greater acceptance and integration in critical decision-making workflows.

The 14 major challenges discussed, benchmarking limitations, prompt sensitivity, uncertainty in interpretation, bias-induced variability, and impact of the pooling mechanism, underscore why LLM-based sentiment analysis remains highly variable and difficult to evaluate consistently. In summary, addressing these challenges requires the following actions.

- To improve stability-aware sentiment evaluation metrics and mitigation strategies.
- To standardize prompt design frameworks to reduce sensitivity.
- To improve interpretability by means of uncertainty-aware training techniques.
- To integrate bias mitigation strategies and domain-adaptive fine-tuning.
- To achieve consensus through ensemble approaches among LLMs in sentiment analysis.
- To achieve the reproducibility and stability in sentiment analysis
- To apply knowledge distillation to mitigate MVP in LLMs for Sentiment Analysis, to obtain SLMs.
- To ensure consistency and robustness in ESA-CDM via prompt-based LLMs
- To enhance interpretability, reliability, and trustworthiness in sentiment analysis with the integration of XAI.
- To promote open source development with reproducibility-aware practices, including shared inference protocols, model cards, and behavioral validation during compression and fine-tuning.

By implementing these solutions, the reliability and trustworthiness of LLM-based sentiment analysis can be significantly improved, paving the way for more consistent and interpretable AI-driven sentiment models.

## 6 Conclusions

Uncertainty and MVP in LLM emerge from a complex interplay of several factors that significantly influence the performance of sentiment analysis. These include stochastic inference mechanisms, uncertainties embedded within training datasets, architectural biases, and prompt sensitivity. A detailed review of the literature confirms that despite notable advancements, LLMs continue to exhibit instability in sentiment classification. This variability presents significant challenges in high-risk applications that demand exceptional accuracy, reliability, and reproducibility, such as financial analytics, healthcare diagnostics, and strategic business decision-making. These concerns highlight the pressing need for robust and effective mitigation strategies.

Furthermore, the issue of sentiment analysis variability is not exclusive to contemporary deep learning approaches but is also deeply rooted in classical sentiment classification methodologies. As highlighted in Wankhade et al. (2022), long-standing challenges such as ambiguity, sarcasm detection, and domain-specific nuances have historically hindered sentiment classification reliability. Thus, an effective solution to sentiment variability should integrate insights from both traditional lexicon-based approaches and modern deep learning methodologies, leveraging the complementary advantages each offers.

Addressing MVP requires a multidimensional approach that incorporates advanced uncertainty quantification frameworks, structured calibration techniques, and interpretability enhancement strategies. This study underscores the need to systematically address uncertainty and variability to develop stable, interpretable, and trustworthy AI-driven sentiment analysis systems. Integration of uncertainty-aware learning methods, ensemble-based consensus strategies, domain-adaptive fine-tuning techniques, and robust explainability mechanisms is vital to mitigating model variability. Collectively, these approaches promise to enhance the reliability and consistency of sentiment classification, promoting greater acceptance and practical deployment in real-world decision-making contexts.

In summary, this study highlights how MVP, which is based on factors such as prompt design, temperature, alignment procedures, and compression, directly affects the consistency and trustworthiness of the LLM output. These challenges are particularly relevant in sentiment-driven applications, where even minor instability can misguide decision-making. Our analysis underscores the urgency of integrating stability-aware design, structured prompt strategies, and uncertainty quantification into the LLM development cycle. Looking ahead, addressing model variability is not just a research priority, but a practical requirement for responsible AI deployment, especially as LLMs are increasingly used in regulated sectors such as healthcare, finance, and public services. Establishing reproducibility standards, interpretability audits, and calibration protocols will be essential to ensure compliance with emerging governance frameworks and to maintain public trust in LLM technologies.

### 6.1 Novel contributions

This review offers several contributions to the understanding of model variability in LLMs: (1) a 12-factor taxonomy of causes contributing to output inconsistency, (2) a detailed analysis of temperature as a variability amplifier in inference, (3) dual case studies demonstrating MVP in real-world LLMs (GPT-4o and Mixtral 8x22B), and (4) 14 mitigation strategies aligned with explainability and trust frameworks. Together, these provide both theoretical grounding and actionable practices for developing more reliable LLM pipelines.

Future work should prioritize formal benchmarks for output consistency under varied temperature settings, as well as develop general-purpose calibration frameworks for post-training stabilization. As open-source LLMs like DeepSeek, LLaMA, Mistral, or Falcon continue to proliferate, reproducibility standards and compression-aware evaluation tools will be essential in ensuring safe and reliable deployments across industry and academia.

## Footnotes

^1^ https://github.com/ari-dasci/OD-TripR-2020Large

^2^ https://github.com/ggml-org/llama.cpp
